# Alcohol metabolism genes and risks of site-specific cancers in Chinese adults: an 11-year prospective study

**DOI:** 10.1002/ijc.33917

**Published:** 2022-01-20

**Authors:** Pek Kei Im, Ling Yang, Christiana Kartsonaki, Yiping Chen, Yu Guo, Huaidong Du, Kuang Lin, Rene Kerosi, Alex Hacker, Jingchao Liu, Canqing Yu, Jun Lv, Robin G Walters, Liming Li, Zhengming Chen, Iona Y Millwood

**Affiliations:** 1 Clinical Trial Service Unit and Epidemiological Studies Unit (CTSU), Nuffield Department of Population Health, University of Oxford, Oxford, UK; 2 Medical Research Council Population Health Research Unit (MRC PHRU), Nuffield Department of Population Health, University of Oxford, Oxford, UK; 3 Chinese Academy of Medical Sciences, Beijing, China; 4 NCDs Prevention and Control Department, Wuzhong CDC, Suzhou, China; 5 Department of Epidemiology and Biostatistics, School of Public Health, Peking University, Beijing, China

**Keywords:** ADH1B, ALDH2, alcohol, cancer, China

## Abstract

Two genetic variants that alter alcohol metabolism, *ALDH2*-rs671 and *ADH1B*-rs1229984, can modify oesophageal cancer risk associated with alcohol consumption in East Asians, but their associations with other cancers remain uncertain. *ALDH2*-rs671 G>A and *ADH1B*-rs1229984 G>A were genotyped in 150,722 adults, enrolled from ten areas in China during 2004-2008. After 11 years’ follow-up, 9339 individuals developed cancer. Cox regression was used to estimate hazard ratios (HRs) for site-specific cancers associated with these genotypes, and their potential interactions with alcohol consumption. Overall, the A-allele frequency was 0.21 for *ALDH2*-rs671 and 0.69 for *ADH1B*-rs1229984, with A-alleles strongly associated with lower alcohol consumption. Among men, *ALDH2*-rs671 AA genotype was associated with HR of 0.69 (95% CI: 0.53-0.90) for IARC alcohol-related cancers (n=1900), compared with GG genotype. For *ADH1B*-rs1229984, the HRs of AG and AA versus GG genotype were 0.80 (0.69-0.93) and 0.75 (0.64-0.87) for IARC alcohol-related cancers, 0.61 (0.39-0.96) and 0.61 (0.39-0.94) for head and neck cancer (n=196), and 0.68 (0.53-0.88) and 0.60 (0.46-0.78) for oesophageal cancer (n=546). There were no significant associations of these genotypes with risks of liver (n=651), colorectal (n=556), stomach (n=725) or lung (n=1135) cancers. Among male drinkers, the risks associated with higher alcohol consumption were greater among *ALDH2*-rs671 AG than GG carriers for head and neck, oesophageal, and lung cancers (p-interaction<0.02). Among women, only 2% drank alcohol regularly, with no comparable associations observed between genotype and cancer. These findings support the causal effects of alcohol consumption on upper aero-digestive tract cancers, with *ALDH2*-rs671 AG genotype further exacerbating the risks.

## Introduction

Cancer is a leading cause of premature mortality and disability globally, accounting for an estimated 19.3 million new cancer cases and 10 million deaths in 2020.^1^ Worldwide, 24% of total cancer cases, including 37% of lung cancer and 47% of digestive tract (oesophagus, stomach, liver) cancers, occurred in China.^1^ Based mainly on observational studies, the International Agency for Research on Cancer (IARC) reported that there is sufficient evidence that alcohol consumption is causally related to development of cancers in the head and neck, oesophagus, liver, colon-rectum, and female breast, but causal evidence remains inconclusive for other cancer sites including lung and stomach due to other possible confounders (e.g. smoking, diet).^2^ Worldwide, it was estimated that 3 million deaths could be attributed to alcohol consumption, including >0.4 million from cancer.^3^

Alcohol consumption has been increasing over recent decades in China, almost exclusively in men,^4^ and is a major contributor to the total cancer burden in Chinese men.^5,6^ In China, and other East Asian populations, two common genetic variants affect alcohol tolerability and are strongly associated with lower alcohol intake.^7^ An East Asian-specific loss-of-function variant in the aldehyde dehydrogenase 2 (*ALDH2*) gene (rs671 G>A) substantially decreases the breakdown of acetaldehyde, which is a Group 1 human carcinogen classified by IARC and a toxic metabolite produced during alcohol metabolism, causing the characteristic East Asian alcohol flushing response.^2,8^ Another variant in the alcohol dehydrogenase 1B (*ADH1B*) gene (rs1229984 G>A) accelerates acetaldehyde formation from alcohol.^9^ These genetic variants, which are randomly allocated at conception and usually independent of other lifestyle exposures, can be used as instruments for alcohol intake to help assess the likely causal effects of alcohol consumption on disease risks.^7,10^ Importantly, appropriate understanding of the interplay between these genetic variants and between genetic variants and alcohol consumption may provide insight into the involvement of alcohol-derived acetaldehyde in the carcinogenesis of certain site-specific cancers. Previous studies have shown that the *ALDH2*-rs671 AA and *ADH1B*-rs1229984 AA and AG genotypes were associated with lower oesophageal cancer risk compared with the GG genotype,^11,12^ and that *ALDH2-*rs671 genotype may modify the relationship between alcohol intake and oesophageal cancer risk.^12-14^ However, there is limited evidence on the associations of these genotypes with risk of cancer at other sites,^11,15^ and for the potential interactions between genotype and alcohol intake on cancer risks.^16-23^ A comprehensive assessment of the interplay between *ALDH2*-rs671, *ADH1B*-rs1229984, and alcohol consumption on risks of different cancer types in a large-scale population-based cohort study may provide valuable insights into the aetiological role of alcohol on different cancers.

Using data from the prospective China Kadoorie Biobank (CKB), we investigated the associations of *ALDH2*-rs671 and *ADH1B*-rs1229984 with total and common site-specific cancers in 151,000 Chinese adults. In addition, we investigated possible gene-alcohol and gene-gene interactions on cancer risks.

## Methods

### Study population

Details of the CKB study design and methods have been previously reported.^24^ Briefly, 512,726 adults aged 30-79 years were recruited from ten rural and urban areas across China during 2004-2008. Trained health workers administered a laptop-based questionnaire recording socio-demographic factors, lifestyles (e.g. alcohol drinking, smoking, diet, physical activity) and medical history; undertook physical measurements (e.g. blood pressure, anthropometry); and collected a blood sample for long-term storage. Two resurveys of ~5% randomly selected surviving participants were conducted using similar procedures in 2008 and 2013-2014. For the baseline survey, ethical approval was obtained from the Ethical Review Committee of the Chinese Centre for Disease Control and Prevention (Beijing, China, 005/2004) and the Oxford Tropical Research Ethics Committee, University of Oxford (UK, 025-04). All participants provided written informed consent.

### Assessment of alcohol consumption

Detailed questionnaire assessment of alcohol consumption has been described previously.^25-27^ Based on their past and current drinking history, participants were classified into: abstainers; ex-regular drinkers; occasional drinkers; and current regular drinkers (i.e. had drunk alcohol in most weeks in the past year). Current regular drinkers were asked further questions about their drinking patterns including drinking frequency, beverage type and amount consumed for each type on a typical drinking day, age started drinking, and experience of alcohol flushing response after drinking. Level of alcohol consumption was calculated as grams (g) of alcohol per week based on frequency, beverage type and amount consumed. Further details of alcohol assessment are reported in the Supplementary Methods.

### Follow-up and main outcome measures

The vital status of participants was obtained periodically from local death registries, supplemented by annual active confirmation through local residential, health insurance, and administrative records. Incident cancers were collected through linkage with cancer registries and the national health insurance system (>98% coverage across the ten study areas), supplemented by active follow-up approach (see Table S1 and Supplementary Methods for further details on completeness and quality of cancer outcome measures).^28^ All events were coded with International Classification of Diseases, 10^th^ Revision (ICD-10), blinded to the baseline information.

The main cancer outcomes investigated in this study were total cancer; IARC alcohol-related cancers (defined as cancers with convincing causal relevance with alcohol as concluded by IARC^2^) which were cancers of the head and neck (included cancers of the lip and oral cavity, pharynx, and larynx; ICD-10: C00-C14, C32), oesophagus (C15), colon-rectum (C18-C20), liver (C22), and female breast (C50); and certain other site-specific cancers including lung cancer (C33-C34) and stomach cancer (C16). Other cancers, apart from ill-defined neoplasms (C76-C80, C97), were combined as “other cancers of known sites”. Upper aero-digestive tract cancers were defined as cancers of the head and neck and oesophagus. By 1 January 2018, 49,459 (9.7%) deaths were recorded among the 512,726 CKB participants, with 5302 (1.0%) lost to follow-up.

### Genotyping

The two variants of interest, *ALDH2*-rs671 and *ADH1B*-rs1229984, were both genotyped in 151,035 randomly selected participants from the CKB cohort using the Affymetrix Axiom® 800K-single nucleotide polymorphism (SNP) array (n=100,168) or 384-SNP Illumina® GoldenGate array (n=92,958) at BGI (Shenzhen, China). Genotyping concordance for the studied variants was high between the two arrays (>99.9% among ~25,400 participants genotyped with both arrays).^7^

### Statistical analysis

Participants with missing data on genomic principal components (n=313) were excluded from the analyses, leaving 150,722 participants in the study (see Figure S1). Means and percentages of baseline characteristics were calculated by genotype, standardised to the age and study area structure of the genotyped study population. Cox proportional hazard models, stratified by age-at-risk and study area and adjusted for 12 genomic principal components, were used to estimate hazard ratios (HRs) for cancers reported during follow-up associated with *ALDH2*-rs671 and *ADH1B*-rs1229984 genotypes, in men and women separately. The genotypic associations with cancer risk were further examined separately by drinking status.

Potential effect modification of the associations between amount of alcohol consumption and cancer risks by genotype were investigated among current regular drinkers. The joint effects of alcohol consumption and *ALDH2*-rs671 were assessed by estimating the HRs associated with four categories defined by baseline alcohol intake (<280, 280+ g/week in men; <70, 70+ g/week in women) and genotype (GG, AG), excluding AA individuals as few of them drank. The models were stratified by age-at-risk and study area and adjusted for 12 genomic principal components, education, household income, smoking, fruit intake, physical activity, body mass index (BMI), family history of cancer. Likelihood ratio test was used to test for interaction between alcohol consumption and genotype by comparing two models with and without the interaction term. As tobacco smoking also produces acetaldehyde, subgroup analyses by smoking status were conducted to assess potential residual confounding from smoking. The joint effects of alcohol and *ADH1B*-rs1229984 were assessed using similar methods. Alcohol intake was also modelled as a continuous variable to estimate adjusted HRs of cancers associated with a 280 g/week higher usual alcohol intake by genotype, with heterogeneity in effect sizes assessed by chi-squared tests.

The joint effects of *ALDH2*-rs671 and *ADH1B*-rs1229984 were examined by estimating the HRs associated with the nine groups defined by the combination of genotypes for both variants (from **GG**/GG to **AA**/AA [***ALDH2*** **-rs671**/*ADH1B*-rs1229984], which represented the highest to lowest mean alcohol intake) in men, stratified by age-at-risk and study area and adjusted for 12 genomic principal components.

Various sensitivity analyses were performed, including: (a) additional adjustments for socio-economic status and major lifestyle risk factors for cancer; (b) area-stratified analysis by combining within-area genotypic effects using inverse variance-weighted meta-analysis to investigate potential residual confounding by population stratification (given the differences in allele frequencies between study areas); and (c) excluding individuals with a prior history of cancer at baseline from the gene-alcohol interaction analysis to reduce potential reverse causation due to changes in drinking habits for health reasons.

As few women drank alcohol regularly in CKB,^25,26^ the main analyses were focused among men, with genotypic analyses among women conducted to assess the presence of pleiotropic effects (i.e. genotypic associations that are not mediated by alcohol consumption). For analyses involving more than two exposure categories, the floating absolute risk method was used to compute the group-specific 95% confidence intervals (CIs) derived from the variance of the log hazard of each category, such that each HR (including the one for the reference group) has a group-specific 95% CI that facilitates comparisons between any two categories, as described previously.^7,29,30^ For comparisons of two groups (i.e. an exposure category with the reference group), conventional 95% CIs were reported. Repeat alcohol measures for participants who attended both subsequent resurveys were used to correct for regression dilution bias.^31^ Further details of the statistical analysis are reported in the Supplementary Methods. All analyses used SAS (version 9.4) and R (version 4.0.4).

## Results

Among the 150,722 study participants, the mean age was 52.1 (SD 10.7) years, 40% were men and 56% lived in rural areas. The overall A-allele frequency was 0.21 (range by area from 0.13 to 0.29) for *ALDH2*-rs671 and 0.69 (from 0.64 to 0.74) for *ADH1B*-rs1229984, with a generally higher frequency in southern than northern areas for both variants (Table S2).

Among men, *ALDH2*-rs671 was strongly associated with the prevalence of current regular drinking (46%, 17%, 1% for GG, AG, and AA, respectively) and mean alcohol intake (143, 35, 2 g/week, respectively) (all *P*_trend_<0.0001) (Table 1). *ADH1B*-rs1229984 genotype was also associated with current regular drinking prevalence (43%, 34%, 32%) and mean alcohol intake (146, 99, 91 g/week). Among male current regular drinkers, *ALDH2*-rs671 was strongly associated with the alcohol flushing response (11%, 56%, 62%) and age at drinking onset (28, 32, 40 years); the correlations with the alcohol flushing response were consistent directionally for A alleles of both variants, but the effects were weaker with *ADH1B*-rs1229984 (15%, 18%, 20%) (*P*_trend_<0.0001 for all above). In women, similar patterns of associations between drinking patterns and genotype were observed as in men, but with very low prevalence of regular drinking (2%) the differences were small in magnitude (Table S3). There were no material effects of these genotypes on smoking or other lifestyle characteristics in men or women, except a slightly higher prevalence of daily fresh fruit intake and lower physical activity in male *ALDH2*-rs671 A-allele carriers, and slightly lower mean BMI in A-allele carriers for both variants in both sexes.

**Figure 1:**
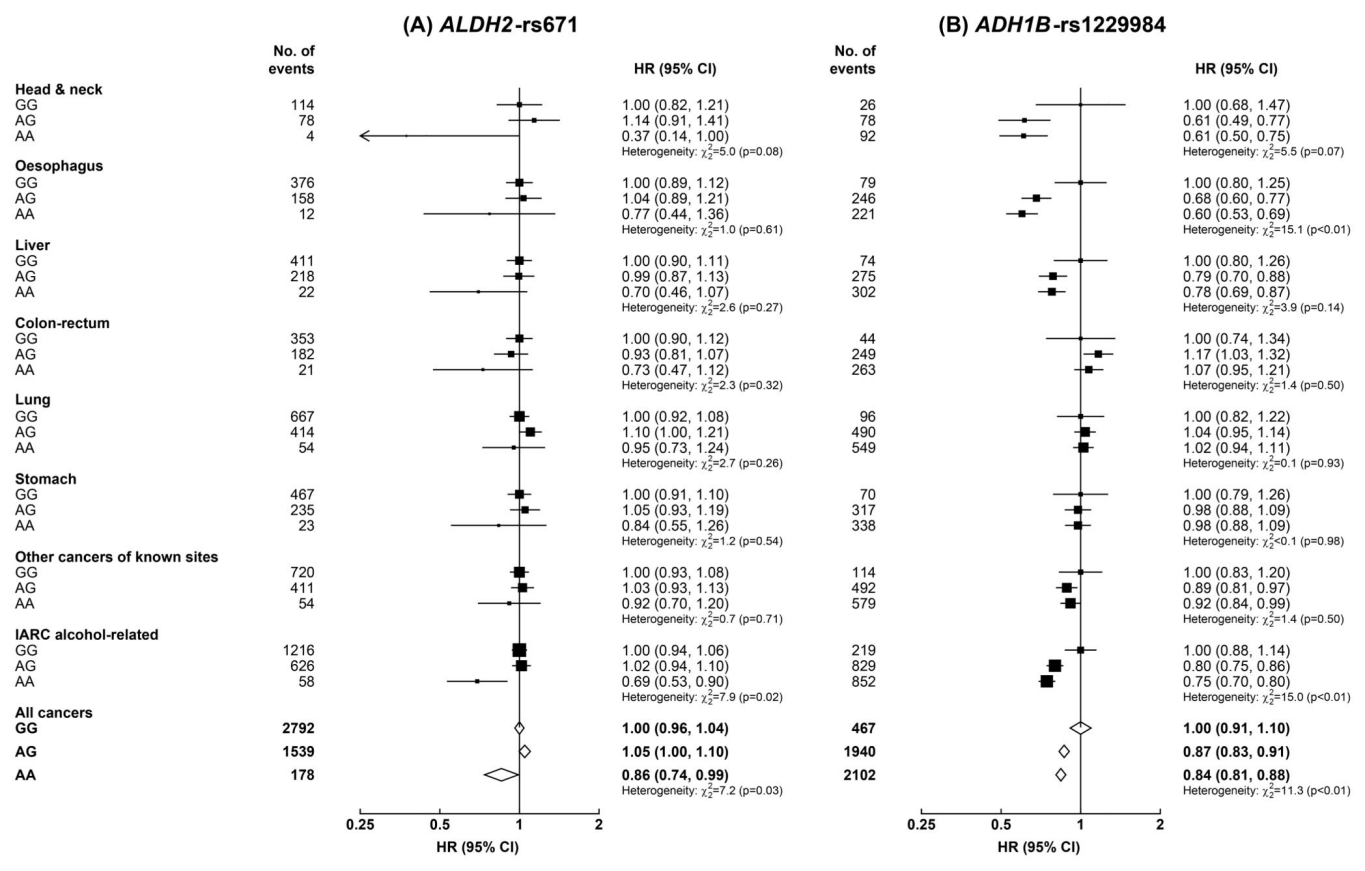
Associations of genotypes for *ALDH2*-rs671 and *ADH1B*-rs1229984 with risks of total and selected site-specific cancers, in men Cox models were stratified by age-at-risk and study area, and adjusted for 12 genomic principal components. Each solid square represents HR with the area inversely proportional to the “floated” variance of the group-specific log hazard. The horizontal lines indicate group-specific 95% CIs. Open diamonds represent the overall HRs for all cancers. IARC alcohol-related cancers included cancers of the head and neck, oesophagus, liver, and colon-rectum. IARC, International Agency for Research on Cancer; HR, hazard ratio; CI, confidence interval.

During a median of 11.2 (interquartile range: 10.3-12.2) years of follow-up, 9339 participants (4509 men, 4830 women) developed cancer. Among men, those with *ALDH2*-rs671 AA genotype had 14% lower risk of any cancer (HR=0.86 [95% CI: 0.73-1.00]) and 31% (0.69 [0.53-0.90]) lower risk of IARC alcohol-related cancers than those with GG genotype (Figure 1A). The associations were directionally consistent, albeit with wide CIs due to the small number of cases involved, for individual IARC alcohol-related cancer sites and for stomach cancer, but not for lung cancer. There were no clear differences in cancer risks between *ALDH2*-rs671 AG and GG genotypes at the overall level, however, the associations appeared to differ by drinking status (Table S4). Compared with *ALDH2*-rs671 GG genotype, AG genotype was associated with significantly higher risks of IARC alcohol-related cancers (1.30 [1.11-1.52]) and oesophageal cancer (2.07 [1.58-2.71]) in male ever-regular drinkers but not in never-regular drinkers. A higher risk of lung cancer was observed in those with AG versus GG genotype among male never-regular drinkers, but not among ever-regular drinkers (Table S4).

For *ADH1B*-rs1229984, compared with men with GG genotype, men with AG or AA genotypes had 13-25% lower risks of overall cancer (0.87 [0.78-0.96], AG vs. GG; 0.84 [0.76-0.93], AA vs. GG) and IARC alcohol-related cancers (0.80 [0.69-0.93]; 0.75 [0.64-0.87]), mainly driven by head and neck cancer (0.61 [0.39-0.96]; 0.61 [0.39-0.94]) and oesophageal cancer (0.68 [0.53-0.88]; 0.60 [0.46-0.78]) (Figure 1B). Men with AG or AA genotypes also tended to have lower, although non-significant, risks of liver cancer, but not of other cancers. The associations of *ADH1B*-rs1229984 with overall cancer, IARC alcohol-related cancers, and oesophageal cancer were directionally consistent across different drinking groups, and were more apparent among ever-regular drinkers (Table S5).

**Figure 2:**
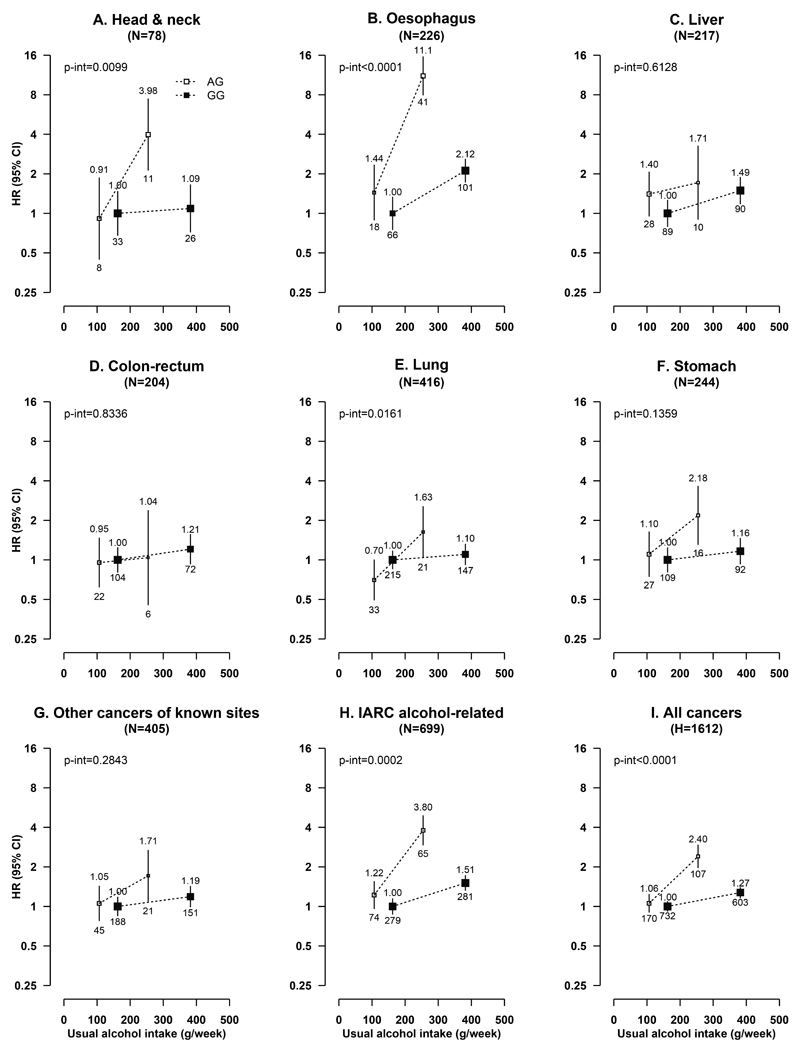
Associations of *ALDH2*-rs671 genotypes with risks of total and selected site-specific cancers at different usual intake levels of alcohol, in male current regular drinkers Cox models were stratified by age-at-risk and study area, and adjusted for 12 genomic principal components, education, household income, smoking status, physical activity, fresh fruit intake, body mass index, and family history of cancer. Each box represents HR with the area inversely proportional to the “floated” variance of the group-specific log hazard. The vertical lines indicate group-specific 95% CIs. The numbers above the error bars are point estimates for HRs, and the numbers below are number of events. Solid boxes denote *ALDH2*-rs671 GG genotype and open boxes denote *ALDH2*-rs671 AG genotype. Alcohol intake, separately in *ALDH2*-rs671 AG and GG drinkers, was classified based on baseline consumption of <280 and ≥280 g/week. AA individuals were excluded as few of them drank (n=28). IARC alcohol-related cancers included cancers of the head and neck, oesophagus, liver, and colon-rectum. IARC, International Agency for Research on Cancer; HR, hazard ratio; CI, confidence interval.

**Figure 3:**
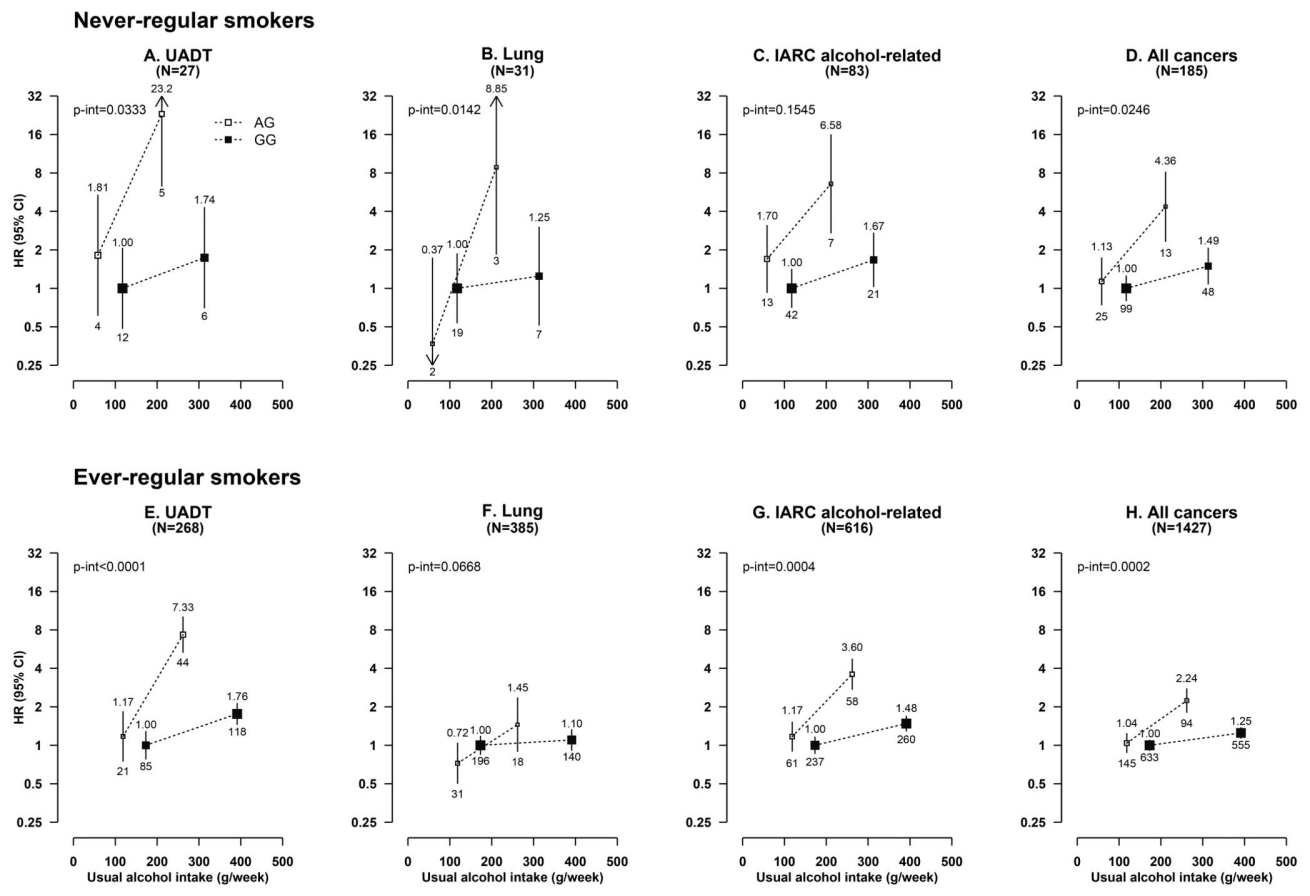
Associations of *ALDH2*-rs671 genotypes with risks of selected cancers at different usual intake levels of alcohol, in male never and ever-regular smokers UADT cancers included cancers of the head and neck and oesophagus. IARC alcohol-related cancers included cancers of the head and neck, oesophagus, liver, and colonrectum. UADT, upper aero-digestive tract; IARC, International Agency for Research on Cancer; HR, hazard ratio; CI, confidence interval. Conventions are as in Figure 2.

Among male current regular drinkers, there was evidence of interactions between alcohol consumption and *ALDH2*-rs671, especially the AG genotype, on risks of overall and IARC alcohol-related cancers. For total cancer, the adjusted HRs were 1.00 (0.92-1.08) for GG and 1.06 (0.91-1.23) for AG drinkers consuming <280 g/week alcohol, and were 1.27 (1.17-1.39) for GG and 2.40 (1.98-2.92) for AG drinkers consuming at least 280 g/week (*P*_interaction_<0.0001); for IARC alcohol-related cancers, the corresponding HRs were more extreme (*P*_interaction_=0.0002). Similar significant interactions between alcohol and *ALDH2*-rs671 were also observed for site-specific cancers, especially oesophageal cancer (*P*_interaction_<0.0001) and head and neck cancer (*P*_interaction_<0.01), and less so for lung cancer (*P*_interaction_=0.016) (Figure 2). The associations between these cancers and joint alcohol-*ALDH2*-rs671 groups were broadly similar in never-regular smokers and in ever-regular smokers (Figure 3). There were no clear interactions between alcohol consumption and *ALDH2*-rs671 for liver or colorectal cancers, but the dose-response association between alcohol and stomach cancer appeared stronger in *ALDH2*-rs671 AG drinkers than in GG drinkers (HR=3.36 [1.73-6.54] vs. 1.02 [0.73-1.41], per 280 g/week; *P*_heterogeneity_=0.002) (Figure S2). For *ADH1B*-rs1229984, no clear interactions with alcohol consumption on cancer risks were observed among male current regular drinkers (Figures S3-S4).

Examination of the joint effects of the two genetic variants in men showed that the risks of IARC alcohol-related cancers and of upper aero-digestive tract cancers were highest for the combination of *ALDH2*-rs671 AG with *ADH1B*-rs1229984 GG genotypes, followed by the combination of GG/GG genotypes, and were lowest for the combinations of *ALDH2-*rs671 AA with *ADH1B*-rs1229984 AG or AA genotypes (Table S6). However, these modest gene-gene interactions were not significant.

In men the genotypic associations with cancers were unaltered with additional adjustment for other cancer risk factors (education, household income, smoking, fresh fruit intake, physical activity, BMI, family cancer history, hepatitis B virus [HBV] infection status) (Figure S5) or in area-stratified as opposed to area-adjusted analyses (Figure S6). Similar *ALDH2*-rs671-alcohol interactions were observed as in the main analyses after excluding individuals with prior cancer or further adjusting for HBV infection status (Figures S7-S8).

**Figure 4:**
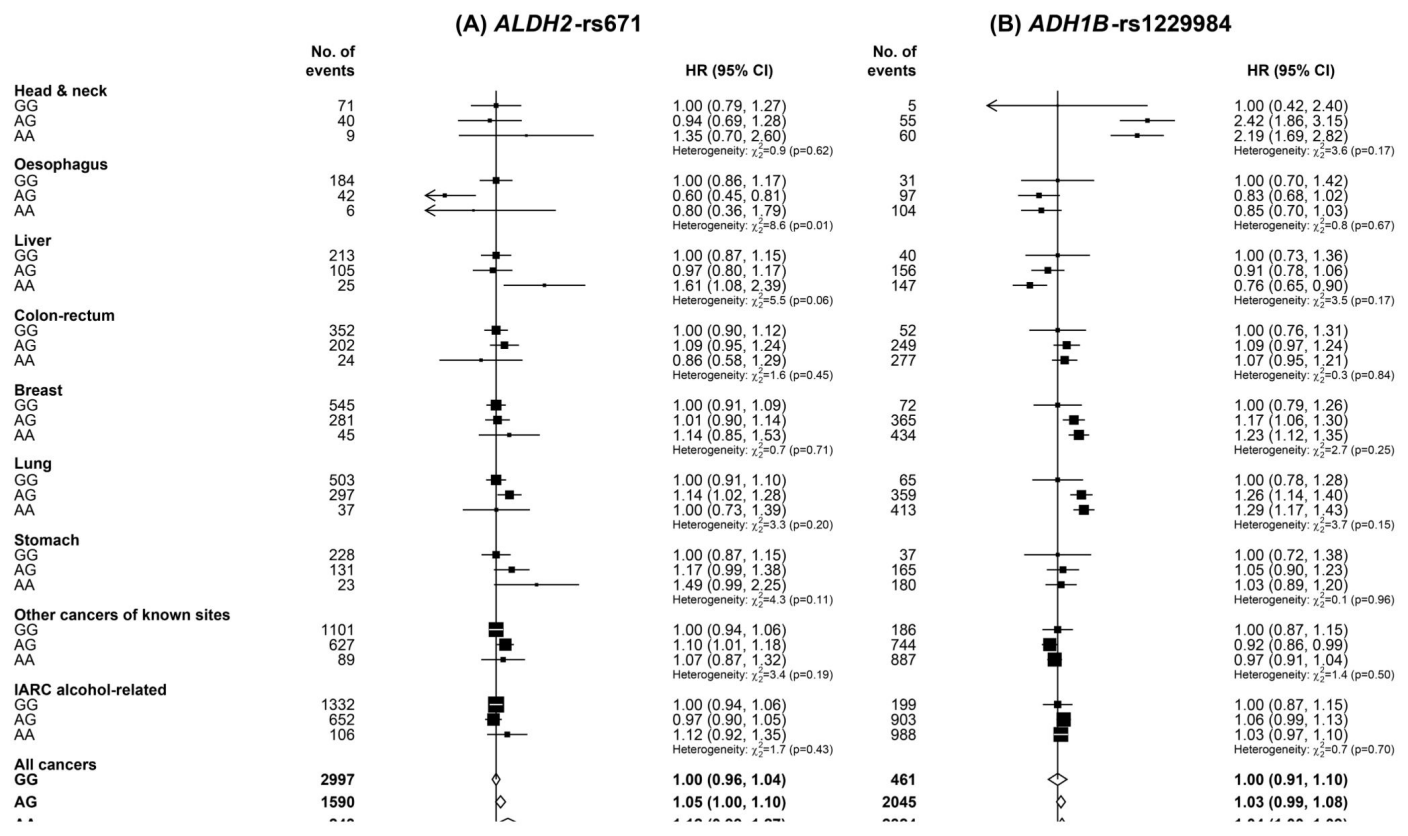
Associations of genotypes for *ALDH2*-rs671 and *ADH1B*-rs1229984 with risks of total and selected site-specific cancers, in women IARC alcohol-related cancers included cancers of the head and neck, oesophagus, liver, colon-rectum, and breast. IARC, International Agency for Research on Cancer; HR, hazard ratio; CI, confidence interval. Conventions are as in Figure 1.

Among women, there were no clear associations of these two genetic variants with risks of overall or IARC alcohol-related cancers (Figure 4). For site-specific cancers, compared with *ALDH2*-rs671 GG genotype, AG genotype was associated with a lower oesophageal cancer risk, while AA genotype was associated with a higher liver cancer risk, but the numbers of cases involved were small. When comparing the associations of these genotypes with cancer risks between men and women, heterogeneity of the associations was seen for several IARC alcohol-related cancers (Table S7). Among women, no clear gene-alcohol interactions were observed (Tables S8-S10).

## Discussion

In this large genetic study of Chinese adults, two common genetic variants, *ALDH2*-rs671 G>A and *ADH1B*-rs1229984 G>A, which strongly reduced alcohol consumption, were associated with lower risks of overall and IARC alcohol-related cancers, especially upper aero-digestive tract cancers, in men among whom over a third drank alcohol regularly. Among male drinkers, *ALDH2*-rs671 genotype significantly modified the effects of alcohol consumption on certain cancers, with greater excess risks in men with the AG than GG genotype for a given level of alcohol consumption, especially for upper aero-digestive tract cancers and potentially for lung cancer, regardless of smoking status. Among women, very few drank alcohol regularly and these variants were not associated with overall or IARC alcohol-related cancer risk.

Previous case-control studies and meta-analyses in East Asian populations have reported associations of *ALDH2*-rs671 genotypes with risks of oesophageal cancer^10,12,32^ and head and neck cancer,^33^ and that the relationships may be modified by alcohol consumption. Compared with GG genotype, AA genotype was associated with an overall lower risk, while AG genotype was associated with a higher risk among drinkers but not among never drinkers.^10,12,33^ The increased risks among *ALDH2*-rs671 AG drinkers, who have markedly elevated acetaldehyde levels after consuming alcohol, but not among never drinkers suggest that acetaldehyde may be the underlying mechanism through which alcohol consumption increases upper aero-digestive tract cancer risk. In the present prospective study of Chinese population, we observed broadly similar effect modifications of *ALDH2*-rs671 genotypes on risks for IARC alcohol-related and oesophageal cancers associated with alcohol consumption, as in previous studies.^10,12,14,33^ For other cancers sites, existing genotypic evidence has been inconclusive.^15,16,19,22,34-38^ A previous meta-analysis of case-control studies found no clear associations of *ALDH2*-rs671 with cancers in the liver (1522 cases, 7 studies), colon-rectum (2356 cases, 10 studies), stomach (5558 cases, 8 studies), or lung (1105 cases, 2 studies).^15^ In contrast, recent large case-control studies (>1000 cases each) in South Korea and Japan showed that *ALDH2*-rs671 AA and AG genotypes were associated with 22-30% lower risk of colorectal cancer,^16^ but approximately 23-30% higher risk of stomach cancer,^19,38^ compared with GG genotype. In CKB, there were generally lower risks of liver, colorectal, and stomach cancers comparing *ALDH2*-rs671 AA versus GG genotype among men, but precision was low due to the small numbers of AA carriers, and no clear genotypic associations were observed for lung cancer.

For *ADH1B*-rs1229984, a meta-analysis of case-control studies involving mainly East Asian populations showed that both AG and AA genotypes were associated with reduced risks of oesophageal cancer (9117 cases, 23 studies) and head and neck cancer (6646 cases, 16 studies) compared with GG genotype.^11^ However, most previous studies found no significant associations of *ADH1B* genotype with other cancer sites.^11,16,19,35,39^ Similarly, in our study we found clear inverse associations of the *ADH1B*-rs1229984 A-allele with risks of overall and IARC alcohol-related cancers, mainly driven by upper aero-digestive tract cancers and potentially also by liver cancer, but there were no clear associations with other cancer sites.

Using genetic variants as proxy for alcohol intake, the inverse associations between genotypes that predict lower alcohol consumption (ALDH2-rs671 AA, *ADH1B*-rs1229984 AA and AG) and IARC alcohol-related cancers among men in CKB, especially upper aero-digestive tract cancers, supported the causal effects of alcohol consumption on these cancers, as consistent with existing observational evidence.^2,5,40^ Observational studies have also linked heavy drinking to excess risks of liver and colorectal cancers, and less consistently of stomach and lung cancers.^2,40^ Taking together our findings for both genetic variants, the non-significant lower risks of liver cancer consistently observed for A-alleles of both variants might suggest causal effects of alcohol consumption, but there was no clear evidence supporting causal relationships of alcohol consumption with colorectal, stomach, or lung cancers. Our findings were broadly consistent with a Mendelian randomisation (MR) study in European ancestry populations using data from ~90 SNPs (without *ALDH2*-rs671) associated with alcohol consumption.^41^ This study showed non-significant positive associations of genetically predicted alcohol consumption with oesophageal cancer and head and neck cancer risk but otherwise found no evidence supporting a causal role of alcohol consumption on other site-specific cancers, which may be due in part to the weak genetic instruments used (variance in alcohol consumption explained, ~0.3%).^41^ In our study, the effects of *ALDH2*-rs671 genotype on upper aero-digestive tract cancers in different drinking groups were influenced by a potential gene-environment interaction between alcohol intake and genotype. This demonstrated the limitations of applying *ALDH2*-rs671 directly in MR studies of alcohol and cancer without careful consideration of possible gene-alcohol interactions. Nevertheless, the strong effect of *ALDH2*-rs671 on alcohol consumption indicates that *ALDH2*-rs671 is a much stronger genetic instrument for alcohol intake in East Asians than those available for MR studies in European ancestry populations. Importantly, appropriate analysis and interpretation of *ALDH2*-rs671-alcohol interactions offers the opportunity to assess the causality of alcohol and to investigate the role of alcohol-derived acetaldehyde in the aetiology of certain cancers.

Previous studies have reported interactions between *ALDH2*-rs671 and alcohol consumption on upper aero-digestive cancer risk in East Asian populations.^12-14,20,23,33^ A meta-analysis of 31 case-control studies involving 8510 cases showed that the ORs of oesophageal cancer comparing *ALDH2*-rs671 AG versus GG genotype increased from 1.21 (0.95-1.73) in non-/rare drinkers to 3.79 (3.05-4.72) in light drinkers and 6.50 (5.34-7.92) in heavy drinkers.^12^ Similar findings have also been reported for head and neck cancer in a meta-analysis of six Japanese case-control studies (945 cases).^33^ In contrast, most existing studies found no evidence of interaction between *ALDH2*-rs671 and alcohol consumption for colorectal cancer,^16,18,23,35,42^ whereas findings for liver cancer,^22,43,44^ stomach cancer,^19,23,38,45-47^ and lung cancer^36,48^ have been inconclusive. In the present prospective study, in addition to the significant *ALDH2*-rs671-alcohol interactions on head and neck cancer and oesophageal cancer which supported findings from previous studies,^12-14,20,33^ there was also suggestive evidence of an *ALDH2*-rs671-alcohol interaction on lung cancer risk, which was concordant with a previous Japanese case-control study (505 cases)^36^ and our previous report of a stronger dose-response association of alcohol with lung cancer among male drinkers reporting the alcohol flushing response.^5^ While we found no clear evidence of interactions between *ALDH2*-rs671 and alcohol consumption for colorectal or liver cancers, the somewhat stronger dose-response association of alcohol intake with stomach cancer among male *ALDH2*-rs671 AG drinkers was consistent with case-control studies in Japan (1375 cases).^19^ In contrast to the *ALDH2*-rs671-alcohol interactions observed, we found no clear interactions between alcohol consumption and *ADH1B*-rs1229984, or between the two genetic variants, on cancer risks, which were largely consistent with previous studies.^16-21,44,49-51^

The biological pathways via which alcohol consumption may cause cancers are not fully understood and likely vary by cancer site. A major pathway proposed is via local exposure to alcohol-formed acetaldehyde, especially in the upper gastrointestinal tract where, in contrast to the liver, the mucosa has limited capacity to eliminate acetaldehyde.^2,52^ After drinking, local acetaldehyde exposure in the upper digestive tract mucosa starts instantly, mainly due to microbial acetaldehyde formation from alcohol in saliva, followed by long-term acetaldehyde formation from alcohol that is diffused back to saliva from blood circulation.^53^ Particularly in *ALDH2*-deficient individuals, excess salivary acetaldehyde may be produced through human ethanol metabolism in the salivary glands, resulting in excess long-term acetaldehyde exposure in the upper digestive tract mucosa.^52,53^ This is supported by our findings of increased upper aero-digestive tract cancer risks only among *ALDH2*-rs671 AG drinkers but not among *ALDH2*-rs671 AG never-regular drinkers, and the greater excess risks in *ALDH2*-rs671 AG drinkers than GG drinkers for a given amount of alcohol consumed. For a given level of alcohol consumption, *ALDH2*-deficient individuals were reported to be exposed to 2-3 fold (salivary) and 5-6 fold (gastric juice) higher acetaldehyde concentrations than those with active ALDH2 enzyme,^52^ supporting the putative involvement of local alcohol-derived acetaldehyde in upper gastrointestinal tract carcinogenesis. In addition to increased alcohol consumption, the *ADH1B*-rs1229984 GG genotype is associated with slower ethanol oxidation such that ethanol remains in the blood and saliva for longer, which may result in prolonged exposure to salivary acetaldehyde due to oral microbial acetaldehyde production from ethanol and consequently increased risks of upper aero-digestive tract cancers.^54,55^ This prolonged salivary acetaldehyde exposure would be greater in the presence of *ALDH2*-rs671 AG genotype.^53^ Alcohol may also increase risks of cancers of the upper digestive and respiratory tract by acting as a solvent for tobacco carcinogens.^40^ Moreover, smoking and heavy drinking combined may also modify the oral microflora to produce higher acetaldehyde levels in saliva.^53^ It is possible that the observed *ALDH2*-rs671-alcohol interactions might be partly related to acetaldehyde from smoking rather than from alcohol consumption alone, especially for lung cancer for which no causality of alcohol has been inferred by genotypic associations. This is however unlikely, as similar *ALDH2*-rs671-alcohol interactions were observed in never-regular smokers, and there were no clear additional excess risks among smokers when we examined the joint effects of alcohol consumption, *ALDH2*-rs671, and smoking on cancer risks, although the number of cases was small (Table S11). It is plausible that in addition to potential interactions between *ALDH2*-rs671 genotype and alcohol in lung cancer risk, these genotypes may interact with other exogenous sources of aldehyde (e.g. air pollution) which could influence risk of lung cancer. Furthermore, experimental studies in mice have shown that ALDH2 expression was detected at various levels in multiple organs other than liver, including lung,^56,57^ and that liver ALDH2 was responsible for clearing only half of circulating acetaldehyde after alcohol intake,^56^ suggesting that multiple *ALDH2*-expressing organs contribute to systemic acetaldehyde clearance. Future studies are warranted to elucidate the potential roles of alcohol and ALDH2 deficiency in the carcinogenesis of lung and other sites. On the other hand, the potential lack of *ALDH2*-rs671-alcohol interaction for liver cancer and colorectal cancer might suggest that other alcohol-induced pathways are more important for carcinogenesis in these sites, e.g. alcohol-induced oxidative stress, changes in folate metabolism, and intestinal inflammation.^2,58^

The chief strengths of this study include the prospective study design, large community-based study population, reliable alcohol consumption data^5,7^ and extensive information on lifestyle risk factors, and reasonably large numbers of incident events for various common cancer sites traced via comprehensive and complete follow-up. We were also able to minimise population stratification bias with adjustments for study area and genomic principal components. Nevertheless, several limitations also warrant consideration. Although the two genetic variants were strong instruments for alcohol intake and were not associated with smoking, they were weakly associated with other cancer risk factors (e.g. fresh fruit intake, physical activity, BMI) in CKB. However, the differences were extremely small in magnitude and might have been the consequences of alcohol consumption. Importantly, additional adjustments for these risk factors did not alter the main findings. Also, the two enzymes affected by the studied genetic variants are involved in many biochemical pathways,^59,60^ which might potentially affect carcinogenesis independent of alcohol consumption. Nonetheless, among women who rarely drank alcohol despite their genotype, there were no clear genotypic associations with IARC alcohol-related cancers or most site-specific cancers. Although *ALDH2*-rs671 was associated with oesophageal cancer and liver cancer among women, which might be partly related to acetaldehyde exposure from other sources (e.g. air pollution, cooking oil fumes, passive smoking) or endogenous aldehyde exposure,^2,59^ the associations were not directionally consistent to those observed in men. These findings suggest the genotypic results in men were likely to be driven chiefly by alcohol consumption rather than by pleiotropic pathways. While further adjustment for HBV infection status did not materially alter our findings, other major risk factors e.g. *Helicobacter pylori* infection for stomach cancer and hepatitis C infection for liver cancer were not available to be included in our analysis. Although these infectious agents are unlikely to be confounding factors in the associations between the studied genetic variants and cancer risks, whether they may interact with alcohol consumption and ALDH2 deficiency remains to be elucidated. Finally, our study may be under-powered to detect any weak causal effects of alcohol intake on site-specific cancers other than upper aero-digestive tract cancers.

In conclusion, in Chinese men *ALDH2*-rs671 G>A and *ADH1B*-rs1229984 G>A genotypes were associated with lower risks of overall and IARC alcohol-related cancers, mainly upper aero-digestive tract cancers. Furthermore, *ALDH2*-rs671 genotype may modify the effects of alcohol consumption on certain cancers, especially upper aero-digestive tract cancers. These findings support the causal role of alcohol consumption in the aetiology of upper aero-digestive tract cancers, which is exacerbated in individuals with inherited low alcohol tolerability. The study reinforces the need to lower population-levels of alcohol consumption for cancer prevention, especially in China where alcohol consumption is increasing despite the low alcohol tolerability among a subset of the population.

## Supplementary Material

Supplementary material

## Figures and Tables

**Table 1:** Baseline characteristics of participants by *ALDH2*-rs671 and *ADH1B*-rs1229984 genotypes, in men

		*ALDH2*-rs671				*ADH1B*-rs1229984			
	Overall (N=60835)	GG (N=38247)	AG (N=19827)	AA (N=2761)	*P*_trend^a^_	GG (N=29078)	AG (N=26025)	AA (N=5732)	*P*_trend^a^_
**Socio-demographic characteristics**									
Mean age, years	52.9	52.9	52.9	53.0	0.11	52.6	53.0	53.0	0.11
Education >6 years, %	57.7	57.8	57.4	56.5	0.053	57.0	57.8	57.6	0.65
Household income >20000 yuan/year, %	44.6	44.6	44.8	44.3	0.84	44.5	44.3	44.9	0.23
**Lifestyle risk factors**									
Current regular smokers, %	60.9	60.9	61.1	59.8	0.38	61.1	60.7	61.1	0.54
Non-daily fresh fruit intake, %	85.1	86.1	83.4	83.4	<0.0001	85.6	85.2	85.0	0.17
Physical activity, mean MET-h/d	22.1	22.2	21.9	21.4	0.030	21.8	22.2	22.0	0.68
Mean body mass index, kg/m^2^	23.5	23.5	23.3	23.2	<0.0001	23.7	23.5	23.4	<0.0001
**Self-reported health and medical history, %**									
Poor self-rated health status	9.0	9.0	8.9	10.0	0.79	9.3	9.1	8.8	0.11
Prior chronic disease^b^	23.0	23.0	23.1	24.0	0.40	22.9	23.4	22.7	0.19
Prior cancer^b^	0.5	0.4	0.5	0.4	0.32	0.5	0.5	0.4	0.52
Family history of cancer	16.9	16.9	16.9	16.7	0.63	17.0	16.7	17.1	0.50
**Alcohol drinking, %**									
Abstainers, %	20.3	9.8	31.2	71.2		15.7	20.4	20.9	
Ex-regular drinkers, %	8.6	10.8	5.8	1.2		9.1	8.2	8.8	
Occasional drinkers, %	37.2	33.3	46.5	26.4		32.1	37.5	38.0	
Current regular drinkers, %	34.0	46.0	16.5	1.2	<0.0001	43.2	33.9	32.3	<0.0001
Mean intake in current drinkers, g/week	286.4	302.7	200.3	89.9	<0.0001	331.9	286.5	275.1	<0.0001
Mean age at drinking onset in current drinkers, year	28.6	28.0	32.1	40.3	<0.0001	28.4	28.7	28.6	0.70
Flushing response in current drinkers, %	18.4	11.4	55.6	61.8	<0.0001	14.6	18.1	19.6	<0.0001
Mean intake overall^c^, g/week	99.4	142.7	35.1	2.4	<0.0001	145.6	99.3	91.0	<0.0001

MET-h/d, metabolic equivalent of task per hour per day. Prevalences and means are adjusted for age (in 10-year intervals) and study areas as appropriate.

a Associations between genotype and baseline characteristics were assessed using multinomial logistic regression for drinking status, logistic regression for binary variables and linear regression for continuous variables, adjusted for age and area where appropriate.

b Based on participants’ self-reported prior disease history at baseline. Prior chronic disease included self-reported history of coronary heart disease, stroke, transient ischaemic attack, diabetes, cancer, tuberculosis, rheumatoid arthritis, peptic ulcer, emphysema/chronic bronchitis, gallstone/gallbladder disease, and kidney disease.

c The overall mean alcohol intake was calculated across all categories of drinking status. Calculations assigned an intake of 0 g/week to baseline non-drinkers, and 5 g/week to baseline occasional drinkers.

## Data Availability

The China Kadoorie Biobank (CKB) is a global resource for the investigation of lifestyle, environmental, blood biochemical and genetic factors as determinants of common diseases. The CKB study group is committed to making the cohort data available to the scientific community in China, the UK and worldwide to advance knowledge about the causes, prevention and treatment of disease. For detailed information on what data is currently available to open access users and how to apply for it, visit: http://www.ckbiobank.org/site/Data+Access. Researchers who are interested in obtaining the raw data from the China Kadoorie Biobank study that underlines this paper should contact ckbaccess@ndph.ox.ac.uk. A research proposal will be requested to ensure that any analysis is performed by bona fide researchers and - where data is not currently available to open access researchers - is restricted to the topic covered in this paper.

## Novelty and impact

Two genetic variants that alter alcohol metabolism, *ALDH2*-rs671 G>A and *ADH1B-rs1229984* G>A, are associated with oesophageal cancer risk in East Asians, but uncertainty remains on their associations with other cancers and potential interactions with alcohol consumption on cancer risks. This large cohort study of Chinese adults showed that both variants were associated with reduced upper aero-digestive tract (UADT) cancer risk. Furthermore, *ALDH2*-rs671 AG genotype exacerbated the effects of alcohol on several cancers, especially UADT cancers.

## Abbreviations

ADH1B: alcohol dehydrogenase 1B
ALDH2: aldehyde dehydrogenase 2
BMI: body mass index
CI: confidence interval
CKB: China Kadoorie Biobank
HBV: hepatitis B virus
HR: hazard ratio
IARC: International Agency for Research on Cancer
ICD-10: International Classification of Diseases, 10th Revision
MR: Mendelian randomisation
SNP: single nucleotide polymorphism
