# Supplementary material for: Alcohol metabolism genes and risks of site‐specific cancers in Chinese adults: An 11‐year prospective study
Source: Int J Cancer. 2022 Jan 20;150(10):1627–39. doi: 10.1002/ijc.33917 (PMC7612513; doi:10.1002/ijc.33917)
Supplement: Supplementary file 1 — Appendix S1 Supporting Information. [file IJC-150-1627-s001.pdf]

Supplementary material

Alcohol metabolism genes and risks of site-specific cancers in Chinese adults: an 11-year prospective study

Pek Kei Im, Ling Yang, Christiana Kartsonaki, Yiping Chen, Yu Guo, Huaidong Du, Kuang Lin, Rene Kerosi, Alex Hacker, Jingchao Liu, Canqing Yu, Jun Lv, Robin G Walters, Liming Li, Zhengming Chen, Iona Y Millwood, on behalf of the China Kadoorie Biobank (CKB) Collaborative Group

Content List of Supplementary Material

Members of the China Kadoorie Biobank Collaborative Group 2

Supplementary Methods 3

Table S1. Distribution of cancer incidents in the CKB by reporting source 7

Figure S1. Flowchart of the study design and selection of study participants 8

Table S2. Genotype distribution and allele frequencies of *ALDH2*-rs671 and *ADH1B*-rs1229984 across the ten study areas 9

Table S3. Baseline characteristics of participants by *ALDH2*-rs671 and *ADH1B*-rs1229984 genotypes, in women 10

Table S4. Adjusted HRs for total and site-specific cancers associated with *ALDH2*-rs671 genotypes in men, stratified by drinking status 11

Table S5. Adjusted HRs for total and site-specific cancers associated with *ADH1B*-rs1229984 genotypes in men, stratified by drinking status 12

Figure S2. Adjusted HRs for total and site-specific cancers per 280 g/week higher usual alcohol intake in male current regular drinkers, stratified by *ALDH2*-rs671 genotype 13

Figure S3. Associations of *ADH1B*-rs1229984 genotypes with risks of total and site-specific cancers at different usual intake levels of alcohol, in male current regular drinkers 14

Figure S4. Adjusted HRs for total and site-specific cancers per 280 g/week higher usual alcohol intake in male current regular drinkers, stratified by *ADH1B*-rs1229984 genotype 15

Table S6. Adjusted HRs for total and site-specific cancers associated with genotypes of both *ADH1B*-rs1229984 and *ALDH2*-rs671, in men 16

Figure S6. Adjusted HRs for total and site-specific cancers associated with genotypes separately for *ALDH2*-rs671 and *ADH1B*-rs1229984 in men, estimated by area-adjusted and area-stratified analyses 18

Figure S7. Associations of *ALDH2*-rs1671 genotypes with risks of total and site-specific cancers at different usual intake levels of alcohol in male current regular drinkers, after excluding individuals with prior cancer at baseline 19

Figure S8. Associations of *ALDH2*-rs1671 genotypes with risks of total and site-specific cancers at different usual intake levels of alcohol in male current regular drinkers, after further adjustment for hepatitis B infection status 20

Table S7. Adjusted HRs for total and site-specific cancers associated with genotypes of *ALDH2*-rs671 and *ADH1B*-rs1229984, stratified by sex 21

Table S8. Adjusted HRs for total and site-specific cancers associated with *ALDH2*-rs671 genotypes in women, stratified by drinking status 22

Table S9. Adjusted HRs for total and site-specific cancers associated with *ADH1B*-rs1229984 genotypes in women, stratified by drinking status 23

Table S10. Adjusted HRs for total and IARC alcohol-related cancers associated with both alcohol intake and genotypes of *ALDH2*-rs671 and *ADH1B*-rs1229984, in female current regular drinkers 24

Table S11. Adjusted HRs for selected cancers associated with both *ALDH2*-rs671 genotypes and usual intake levels of alcohol in male current regular drinkers, stratified by smoking status 25

References 26

Members of the China Kadoorie Biobank Collaborative Group

International Steering Committee: Junshi Chen, Zhengming Chen (PI), Robert Clarke, Rory Collins, Yu Guo, Liming Li (PI), Chen Wang, Jun Lv, Richard Peto, Robin Walters.

International Co-ordinating Centre, Oxford: Daniel Avery, Fiona Bragg, Derrick Bennett, Ruth Boxall, Ka Hung Chan, Yumei Chang, Yiping Chen, Zhengming Chen, Johnathan Clarke; Robert Clarke, Huaidong Du, Zammy Fairhurst-Hunter, Hannah Fry, Simon Gilbert, Alex Hacker, Parisa Hariri, Michael Holmes, Pek Kei Im, Andri Iona, Maria Kakkoura, Christiana Kartsonaki, Rene Kerosi, Kuang Lin, Mohsen Mazidi, Iona Millwood, Qunhua Nie, Alfred Pozarickij, Paul Ryder, Sam Sansome, Dan Schmidt, Rajani Sohoni, Becky Stevens, Iain Turnbull, Robin Walters, Lin Wang, Neil Wright, Ling Yang, Xiaoming Yang, Pang Yao.

National Co-ordinating Centre, Beijing: Yu Guo, Xiao Han, Can Hou, Chun Li, Chao Liu, Jun Lv, Pei Pei, Canqing Yu.

10 Regional Co-ordinating Centres:

Guangxi Provincial CDC: Naying Chen, Duo Liu, Zhenzhu Tang. Liuzhou CDC: Ningyu Chen, Qilian Jiang, Jian Lan, Mingqiang Li, Yun Liu, Fanwen Meng, Jinhuai Meng, Rong Pan, Yulu Qin, Ping Wang, Sisi Wang, Liuping Wei, Liyuan Zhou. **Gansu** Provincial CDC: Caixia Dong, Pengfei Ge, Xiaolan Ren. Maiji CDC: Zhongxiao Li, Enke Mao, Tao Wang, Hui Zhang, Xi Zhang. **Hainan** Provincial CDC: Jinyan Chen, Ximin Hu, Xiaohuan Wang. Meilan CDC: Zhendong Guo, Huimei Li, Yilei Li, Min Weng, Shukuan Wu. **Heilongjiang** Provincial CDC: Shichun Yan, Mingyuan Zou, Xue Zhou. Nangang CDC: Ziyan Guo, Quan Kang, Yanjie Li, Bo Yu, Qinai Xu. **Henan** Provincial CDC: Liang Chang, Lei Fan, Shixian Feng, Ding Zhang, Gang Zhou. Huixian CDC: Yulian Gao, Tianyou He, Pan He, Chen Hu, Huarong Sun, Xukui Zhang. **Hunan** Provincial CDC: Biyun Chen, Zhongxi Fu, Yuelong Huang, Huilin Liu, Qiaohua Xu, Li Yin. Liuyang CDC: Huajun Long, Xin Xu, Hao Zhang, Libo Zhang. **Jiangsu** Provincial CDC: Jian Su, Ran Tao, Ming Wu, Jie Yang, Jinyi Zhou, Yonglin Zhou. Suzhou CDC: Yihe Hu, Yujie Hua, Jianrong Jin Fang Liu, Jingchao Liu, Yan Lu, Liangcai Ma, Aiyu Tang, Jun Zhang. **Qingdao** Qingdao CDC: Liang Cheng, Ranran Du, Ruqin Gao, Feifei Li, Shanpeng Li, Yongmei Liu, Feng Ning, Zengchang Pang, Xiaohui Sun, Xiaocao Tian, Shaojie Wang, Yaoming Zhai, Hua Zhang, Licang CDC: Wei Hou, Silu Lv, Junzheng Wang. **Sichuan** Provincial CDC: Xiaofang Chen, Xianping Wu, Ningmei Zhang, Weiwei Zhou. Pengzhou CDC: Xiaofang Chen, Jianguo Li, Jiaqiu Liu, Guojin Luo, Qiang Sun, Xunfu Zhong. **Zhejiang** Provincial CDC: Weiwei Gong, Ruying Hu, Hao Wang,Meng Wan, Min Yu. Tongxiang CDC: Lingli Chen, Qijun Gu, Dongxia Pan, Chunmei Wang, Kaixu Xie, Xiaoyi Zhang.

Supplementary Methods

Assessment of alcohol consumption

In the baseline questionnaire, participants were asked how often they had drunk alcohol during the past 12 months (never or almost never, occasionally, only at certain seasons, every month but less than weekly, usually at least once a week). Those who had not drunk alcohol at least weekly in the past 12 months were asked if there was a period of at least a year prior to that when they had drunk some alcohol at least once a week. Based on this information, participants were classified into: abstainers (had never drunk alcohol in the past year and had not drunk in most weeks in the past); ex-regular drinkers (had not drunk alcohol in most weeks in the past year but had done so in the past); occasional drinkers (had drunk alcohol but less than weekly in the past year and had not drunk alcohol in most weeks in the past); and current regular drinkers (had drunk alcohol in most weeks in the past year).

Current regular drinkers were asked further questions about their drinking patterns including: frequency of drinking in the past year (1-2, 3-5, or 6-7 days per week); types of beverage (beer, grape wine, rice wine, weak spirits with <40% alcohol content, strong spirits with ≥40% alcohol content) and amount consumed for each beverage type (reported by number of small [250 ml] or large [640 ml] bottles of beer, and number of liang [50 g] for wines and spirits) on a typical drinking day. Total level of alcohol consumption was calculated as grams per week based on the beverage type and amount drunk on a typical drinking day and frequency of drinking (taken as the median of the reported frequency intervals, i.e., 1.5 for 1-2 days/week, 4 for 3-5 days/week, 6.5 for 6-7 days/week), assuming the following alcohol content by volume (v/v) typically seen in China: beer 4%, grape wine 12%, rice wine 15%, weak spirits 38%, and strong spirits 53%.[1] To calculate overall mean alcohol intake, a mean intake of 5 g/week (regardless of past drinking patterns) was assigned to participants who drank sometimes but less than weekly.

Current regular drinkers were also asked questions about the age they started drinking in most weeks, and their experience of flushing or dizziness after drinking (soon after the first mouthful, after drinking a small amount of alcohol, after drinking a large amount of alcohol, no flushing). The alcohol flushing response was defined by the self-reported experience of hot flushes soon after drinking the first mouthful or a small amount of alcohol.

Follow-up for cancer incidence

The vital status of participants was obtained periodically from local death registries, supplemented by annual active confirmation through local residential, health insurance, and administrative records. In addition, incident cancers were collected through linkage, via unique national identification, with cancer registries and the national health insurance system for any episode of hospitalization (>98% coverage across the ten study areas), supplemented by active follow-up approach to minimize loss to follow-up and underreporting of events. All events were coded with International Classification of Diseases, 10th Revision (ICD-10), blinded to the baseline information.

In the CKB, the reporting of incident cancer events from cancer registries covered about 46% of all cancer incidents recorded (**Table S1**). Death registries and the national health insurance system were the main reporting sources of cancer events in the CKB (covering >94% of all cancer incidents), with cancer registries serving as an additional data source. The cancer mortality rate in the CKB has been shown to be consistent with that from the National Central Cancer Registry of China, while the cancer incidence rate was much higher in the CKB[2] because of the comprehensive and complete cancer monitoring via different active and passive systems in the CKB. Ongoing cancer outcome adjudication in a subset of cancer cases via review of medical notes showed a ~90% diagnosis reporting accuracy.

Genotyping

The two variants of interest, *ALDH2*-rs671 and *ADH1B*-rs1229984, were genotyped in 167,734 participants using the Affymetrix Axiom® 800K-single nucleotide polymorphism (SNP) array (n=100,168) or 384-SNP Illumina® GoldenGate array (n=92,958) at BGI (Shenzhen, China). Genotyping concordance for the studied variants was previously shown to be high between the two arrays (>99.9% among ~25,400 participants genotyped with both arrays).[3] Where discordant, genotypes obtained from the Affymetrix Axiom® 800K-SNP array were used. The genotyped population included 151,035 randomly selected participants and an additional 16,699 participants who had been selected as part of nested case-control studies of stroke, coronary heart disease, and chronic obstructive pulmonary disease. To avoid potential selection bias, only the randomly-selected participants were included in the present study.

Statistical analyses

Participants with missing data on genomic principal components (n=313) were excluded from the analyses, leaving 150,722 randomly-selected genotyped participants in the study (see **Figure S1**).

Associations of cancers with individual genetic variant

Cox proportional hazard models were fitted to estimate the HRs for cancers associated with the genotype of *ALDH2*-rs671 (GG [reference group], AG, AA) and of *ADH1B*-rs1229984 (GG [reference group], AG, AA) in all men and women separately. Cox models were stratified by age-at-risk (five-year groups) and study area (ten areas), and adjusted for 12 genomic principal components. The analyses were repeated separately among never-regular drinkers and ever-regular drinkers.

Effect modification on alcohol intake and cancer risk by individual genetic variant

Potential effect modifications of the associations between amount of alcohol intake and cancer risks by genotype were investigated among current regular drinkers. For assessment of the alcohol-cancer associations in relation to *ALDH2*-rs671, participant with *ALDH2*-rs671 AA genotype were excluded from the analysis given that only a few of them were current regular drinkers. To investigate the joint effects between alcohol intake and *ALDH2*-rs671, four exposure groups were created based on the level of baseline alcohol intake (<280, 280+ g/week in men; <70, 70+ g/week in women) and *ALDH2*-rs671 genotype (GG, AG). Cox proportional hazard models, stratified by age-at-risk and study area and adjusted for 12 genomic principal components, education (no formal school, primary school, middle or high school, technical school/college or above), household income (<10 000, 10 000-19 999, 20 000-34 999, 35 000+ yuan/year), smoking (five groups in men: never, occasional, ever regular <15, ever regular 15-24, ever regular 25+ cigarettes equivalent/day; four groups in women: never, occasional, ex-regular, current), physical activity (continuous, in metabolic equivalent of task hours [MET-h] per day), fruit intake (daily vs. less than daily), BMI (<22, 22-24.9, 25-26.9, 27+ kg/m^2), and family history of cancer (yes/no), were used to estimate HRs of cancers for each group (reference group: GG <280 g/week). These covariates were selected based on their relationships with cancer and their correlations with alcohol drinking behaviours reported in existing literature and in CKB.[4-7] To test for heterogeneity in the HRs associated with alcohol intake across genotypes, a likelihood ratio test was used to compare the two models, with and without the interaction term between alcohol intake and genotype. The same approach was used to assess the interaction between alcohol intake and *ADH1B*-rs1229984 genotype (GG, AG/AA). Cox models, adjusted for the same covariates as in the analysis of alcohol-genotype joint effects, were used to estimate adjusted HRs of cancers associated per 280 g/week higher usual alcohol intake (by modelling alcohol intake as a continuous variable), i.e. around four drinks per day. The HRs per 280 g/week higher alcohol intake were examined across genotype for each of the two genetic variants, with heterogeneity in HRs assessed by chi-squared tests.

As tobacco smoking is a source of acetaldehyde, the joint effect analyses were repeated among male never-regular smokers (i.e. never [smoked <100 cigarettes in lifetime] or occasional [ever smoked occasionally but had never smoked regularly, i.e. on most days, in lifetime] smokers) and ever-regular smokers (i.e. ex-regular or current regular smokers) to examine the extent to which the *ALDH2*-rs671-alcohol interaction might be related to residual confounding from acetaldehyde in tobacco smoke. The joint effects of alcohol intake (<280, 280+ g/week), genotype (GG vs. AG for *ALDH2*-rs671; GG vs. AG/AA for *ADH1B*-rs1229984), and smoking (never-regular, ever-regular) were examined by estimating the HRs associated with eight exposure groups created based on these three variables.

Associations of cancers and the joint effects of both genetic variants

To assess the joint effects of the two genetic variants, we estimated the HRs for cancers associated with the nine genotypes defined by the combination of genotypes of both genetic variants (***ALDH2*-rs671**/*ADH1B*-rs1229984, from **GG**/GG [reference group] to **AA**/AA) in all men, with gene-gene interaction tested by a likelihood ratio test as in the analysis of gene-alcohol interaction. Cox models were stratified by age-at-risk and study area and adjusted for 12 genomic principal components.

Standard tests using scaled Schoenfeld residuals and comparison of HRs of the first five and subsequent years of follow-up suggested no clear evidence of violation of the proportional hazard assumption. For analyses involving comparisons of just two groups (i.e. an exposure category with the reference group), conventional 95% CIs were reported. For analyses involving more than two categories of exposure, group-specific 95% CIs of the HRs were estimated using the variance of the log hazard of each category including the reference group, enabling comparison between any two categories (rather than just pairwise comparisons with the reference group) in the tables and figures.[8] For associations with level of alcohol consumption, repeat alcohol measures for participants who attended both subsequent resurveys were used to correct for regression dilution bias.[9] All P values were two-sided and $P < 0.05$ denotes statistical significance.

Adjustment for regression dilution bias

Within-person variation of self-reported alcohol intake was addressed using the regression dilution adjustment approach,[9] based on methodology developed and reported in a previous study.[10] The usual alcohol intake in each exposure category was taken to be the average intake of the two resurveys in 2008 and 2013-2014, assuming that occasional drinkers consumed 5 g/week. The HRs for the joint categories of alcohol intake and genotype were plotted against their corresponding mean usual alcohol intake. The regression dilution ratio (RDR) was calculated using the assumption-free, non-parametric McMahon-Peto method,[11] taken as the ratio of the range (i.e. difference in the mean alcohol intake of the top vs. bottom [i.e. 420+ vs. <140 g/week, in men] baseline-defined groups) of the usual alcohol intake to the range of baseline alcohol intake. For this report, the RDRs calculated using the McMahon-Peto method were 0.53 for men, broadly similar to the estimates obtained from the self-correlation[9] and the Rosner's regression method.[12] Log HR estimates and corresponding SEs for baseline alcohol intake, modelled as a continuous variable, were then divided by the RDR calculated from the McMahon-Peto method to obtain estimated HRs per 280 g/week higher usual alcohol intake among current regular drinkers, assuming a linear association. The HR per 100 g/week is approximately the cube root of the HR per 280 g/week (as log HR per 100 g/week is [100/280] times log HR per 280 g/week).

Sensitivity analyses

Analysis of the genotypic associations with cancers were repeated with further adjustments for major cancer risk factors (education, household income, smoking, physical activity, fresh fruit intake, BMI, family history of cancer, HBsAg). Area-stratified analysis was conducted to investigate potential residual confounding by population stratification (systematic difference in allele frequencies between study areas). This was done by estimating within-area genotypic associations for each study area (each reflecting purely genotypic effects), using Cox models

stratified by age-at-risk and adjusted for the corresponding genomic principal components within study area. The within-area genotypic associations were then combined using inverse-variance-weighted fixed effects meta-analysis to yield the overall genotypic effects in the study population, stratified by study area. Analyses on *ALDH2*-rs671-alcohol interactions were repeated with further adjustment for HBsAg, and by excluding participants with self-reported prior cancer at baseline. All sensitivity analyses did not change the results observed in the main analyses.

Table S1. Distribution of cancer incidents in the CKB by reporting source

	Cancer registries	
	**No**	**Yes**
**Death certificates**		
No	2645 (28%)	1954 (21%)
Yes	2415 (26%)	2325 (25%)
**Health insurance**		
No	1199 (13%)	1299 (14%)
Yes	3861 (41%)	2980 (32%)

Based on the first cancer event reported among the 9339 cancer developing participants in the study.

Figure S1. Flowchart of the study design and selection of study participants

A) Participants in the main analyses

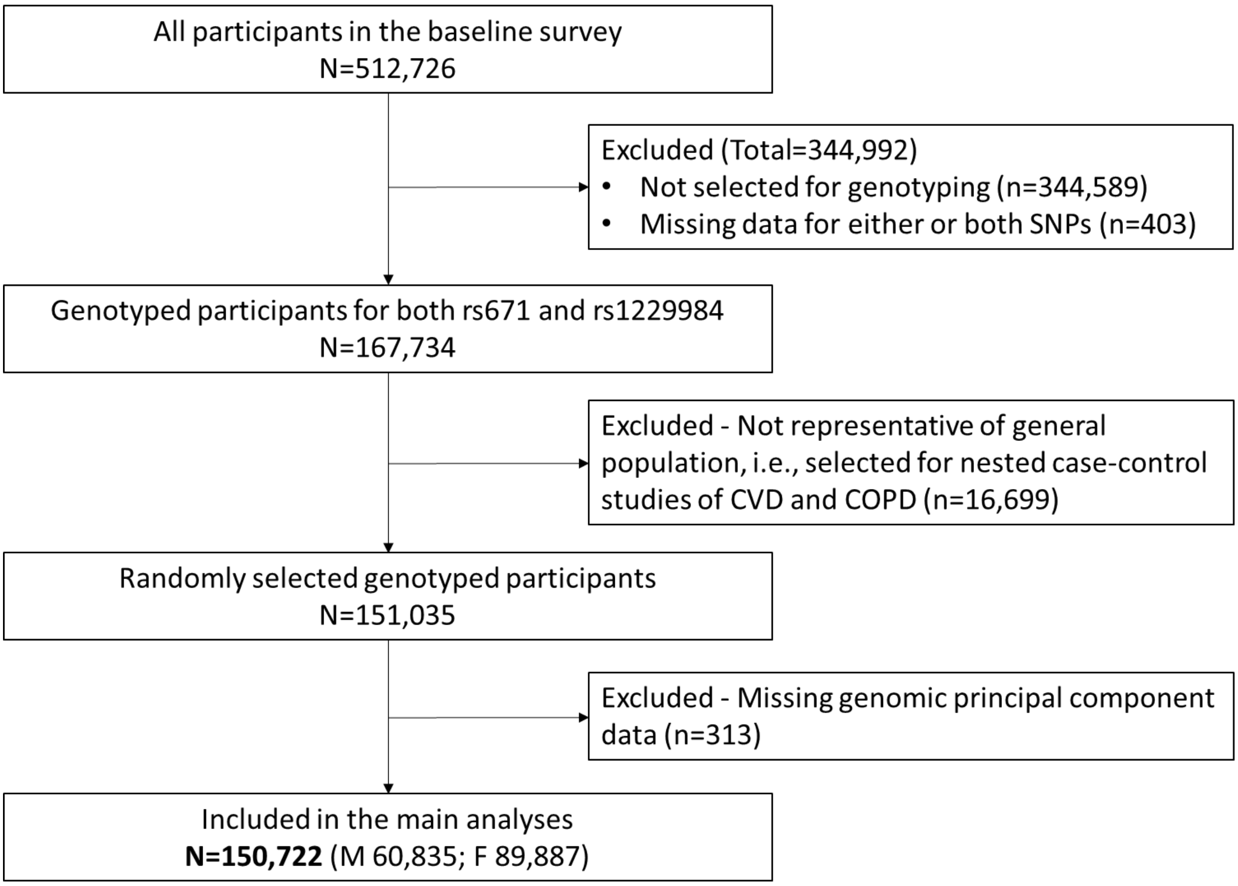

B) Participants in the sensitivity analysis of area-stratified analysis

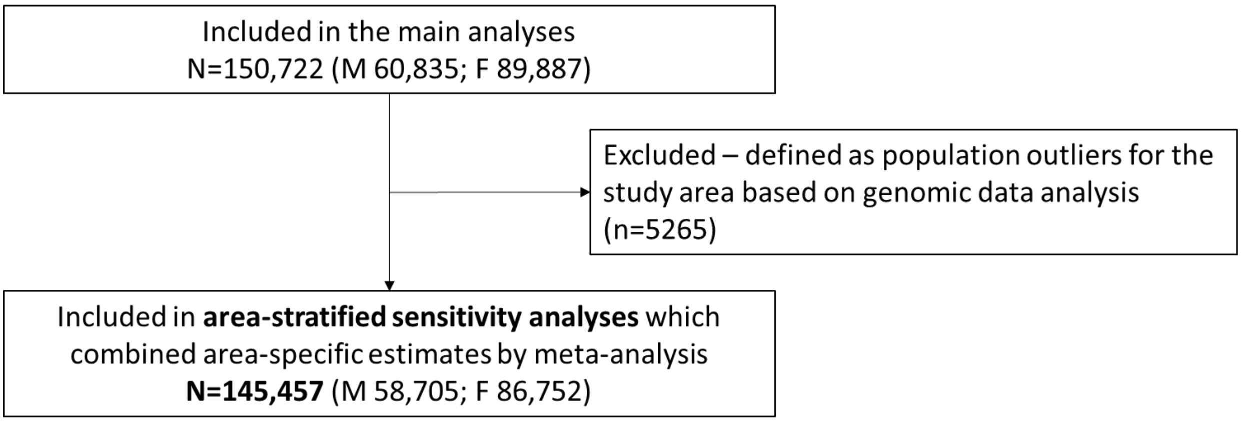

SNP, single nucleotide polymorphism; CVD, cardiovascular disease; COPD, chronic obstructive pulmonary disease.

Table S2. Genotype distribution and allele frequencies of *ALDH2*-rs671 and *ADH1B*-rs1229984 across the ten study areas

		ALDH2-rs671				*ADH1B*-rs1229984			
	**Overall N**	**GG**	**AG**	**AA**	**A-allele frequency[a,b]**	**GG**	**AG**	**AA**	**A-allele frequency[a,b]**
**Study area[c]**									
Harbin (Urban)	17839	12495	4864	480	0.16	1926	7958	7955	0.67
Qingdao (Urban)	11669	7786	3520	363	0.18	1157	5014	5498	0.69
Suzhou (Urban)	15060	8957	5345	758	0.23	1299	6174	7587	0.71
Liuzhou (Urban)	13924	7996	5082	846	0.24	1228	5803	6893	0.70
Haikou (Urban)	7656	3838	3148	670	0.29	538	2922	4196	0.74
Gansu (Rural)	16093	11829	3939	325	0.14	2083	7514	6496	0.64
Henan (Rural)	17760	13390	4073	297	0.13	2022	7981	7757	0.66
Sichuan (Rural)	16377	10705	5114	558	0.19	1686	7063	7628	0.68
Zhejiang (Rural)	18016	9376	7196	1444	0.28	1442	7333	9241	0.72
Hunan (Rural)	16328	8761	6447	1120	0.27	1269	6406	8653	0.73
**All areas**	**150722**	**95133**	**48728**	**6861**	**0.21**	**14650**	**64168**	**71904**	**0.69**

[a] A-alleles decrease alcohol tolerability. Genotype distributions did not deviate from Hardy-Weinberg equilibrium within study areas.
[b] Corresponding frequencies in European-origin populations (1KGP) are 0.00 (*ALDH2*-rs1229984) and 0.03 (*ADH1B*-rs1229984).
[c] Within rural and urban level, the study areas are ordered from North to South.

Table S3. Baseline characteristics of participants by *ALDH2*-rs671 and *ADH1B*-rs1229984 genotypes, in women

		ALDH2-rs671			*ADH1B*-rs1229984		
	**Overall (N=89887)**	**GG (N=56886)**	**AG (N=28901)**	**AA (N=4100)**	**GG (N=42826)**	**AG (N=38143)**	**AA (N=8918)**
**Socio-demographic characteristics**							
Mean age, years	51.5	51.4	51.5	51.9	51.4	51.5	51.4
Education >6 years, %	43.2	43.1	43.3	43.6	42.4	43.0	43.6
Household income >20000 yuan/year, %	39.4	39.6	39.3	39.2	38.8	39.3	39.6
**Lifestyle risk factors**							
Current regular smokers, %	2.4	2.4	2.2	2.6	2.6	2.3	2.3
Non-daily fresh fruit intake, %	77.5	77.6	77.3	76.6	77.7	77.7	77.3
Physical activity, mean MET-h/d	20.5	20.5	20.5	20.5	20.3	20.5	20.5
Mean body mass index, kg/m^2	23.9	23.9	23.8	23.8	24.0	23.9	23.8
**Health and medical history, %**							
Poor self-reported health status	11.5	11.4	11.6	11.5	11.7	11.6	11.3
Prior chronic disease	22.4	22.4	22.6	21.7	22.5	22.3	22.5
Prior cancer	0.5	0.5	0.5	0.7	0.4	0.5	0.5
Family history of cancer	16.7	16.8	16.7	16.9	17.3	16.7	16.7
**Alcohol drinking, %**							
Abstainers, %	63.9	59.2	70.6	87.5	60.9	63.6	64.8
Ex-regular drinkers, %	0.9	1.1	0.4	0.1	0.9	0.9	0.8
Occasional drinkers, %	33.2	36.8	28.2	12.2	35.0	33.4	32.5
Current regular drinkers, %	2.1	2.8	0.8	0.2	3.1	2.1	1.8
Mean intake in current drinkers, g/week	115.3	119.6	86.3	38.1	135.7	114.4	110.0
Age at drinking onset in current drinkers, year	37.3	36.7	41.1	44.1	37.3	37.2	37.3
Flushing response in current drinkers, %	22.7	17.7	57.8	24.4	17.8	22.8	24.6
Mean intake overall[a], g/week	4.1	5.3	1.9	0.7	5.9	4.1	3.7

MET-h/d, metabolic equivalent of task per hour per day.
Prevalences and means are adjusted for age (in 10-year intervals) and study areas as appropriate.
Associations between genotype and baseline characteristics were assessed using logistic regression and linear regression, where appropriate, adjusted for age and area: *P* for trend across genotypes are >0.05 for most socio-demographic, lifestyle and medical history variables, except education (*P*=0.006, *ADH1B*-rs1229984), and BMI (*P*=0.031, *ALDH2*-rs671; *P*=0.005, *ADH1B*-rs1229984); *P* for trend <0.05 for drinking variables, except age at drinking onset by *ADH1B*-rs1229984 (*P*=0.60).
[a] The overall mean alcohol intake was calculated across all categories of drinking status. Calculations assign an intake of 0 g/week to baseline non-drinkers, and 5 g/week to baseline occasional drinkers.

Table S4. Adjusted HRs for total and site-specific cancers associated with *ALDH2*-rs671 genotypes in men, stratified by drinking status

		ALDH2-rs671					
		GG		AG		AA	
	Drinking status	N	HR (95% CI)	N	HR (95% CI)	N	HR (95% CI)
Head and neck	Never-regular	34	1.00 (0.70-1.43)	53	1.28 (0.98-1.67)	4	0.49 (0.18-1.30)
	Ever-regular	80	1.00[a]	25	1.35 (0.86-2.13)	--	--
Oesophagus	Never-regular	173	1.00 (0.83-1.21)	83	0.93 (0.76-1.13)	12	1.25 (0.70-2.23)
	Ever-regular	203	1.00[a]	75	2.07 (1.58-2.71)***	--	--
Liver	Never-regular	156	1.00 (0.84-1.20)	164	1.11 (0.96-1.28)	22	0.82 (0.53-1.25)
	Ever-regular	255	1.00[a]	54	1.02 (0.76-1.38)	--	--
Colon-rectum	Never-regular	122	1.00 (0.82-1.21)	136	1.01 (0.86-1.19)	19	0.74 (0.47-1.16)
	Ever-regular	231	1.00[a]	46	0.96 (0.69-1.31)	--	--
Lung	Never-regular	193	1.00 (0.86-1.17)	312	1.44 (1.29-1.60)***	53	1.27 (0.97-1.67)
	Ever-regular	474	1.00[a]	102	1.06 (0.85-1.31)	--	--
Stomach	Never-regular	197	1.00 (0.84-1.18)	171	1.09 (0.94-1.25)	23	0.99 (0.65-1.50)
	Ever-regular	270	1.00[a]	64	1.21 (0.92-1.60)	--	--
Other cancers of known sites	Never-regular	261	1.00 (0.87-1.14)	313	1.11 (1.00-1.24)	50	0.95 (0.72-1.26)
	Ever-regular	459	1.00[a]	98	1.05 (0.84-1.31)	--	--
IARC alcohol-related	Never-regular	473	1.00 (0.90-1.11)	432	1.07 (0.98-1.17)	56	0.80 (0.62-1.05)
	Ever-regular	743	1.00[a]	194	1.30 (1.11-1.52)**	--	--
All cancers[b]	Never-regular	1017	1.00 (0.93-1.07)	1114	1.16 (1.10-1.23)***	172	1.01 (0.87-1.18)
	Ever-regular	1775	1.00[a]	425	1.19 (1.07-1.32)**	--	--

HR, hazard ratio; CI, confidence interval; IARC, International Agency for Research on Cancer.
Cox models were stratified by age-at-risk and study area, and adjusted for 12 genomic principal components.
Never-regular drinkers included abstainers and occasional drinkers; ever-regular drinkers included ex-regular and current regular drinkers.
Among never-regular drinkers, HRs were presented with group-specific 95% CIs to enable comparison between any two genotypes.
[a] Among ever-regular drinkers *ALDH2*-rs671 AA individuals were excluded from the analysis due to small numbers, and HRs were presented with conventional 95% CIs for two-way comparison of AG vs. GG.
[b] All cancers included ill-defined neoplasm and are patient-based.
*P<0.05; **P<0.01; ***P<0.001, for association comparing the marked genotype versus GG genotype within the same drinking status.

Table S5. Adjusted HRs for total and site-specific cancers associated with *ADH1B*-rs1229984 genotypes in men, stratified by drinking status

		ADH1B-rs1229984					
		GG		AG		AA	
	**Drinking status**	**N**	**HR (95% CI)**	**N**	**HR (95% CI)**	**N**	**HR (95% CI)**
Head and neck	Never-regular	9	1.00 (0.52-1.93)	40	0.69 (0.50-0.94)	42	0.60 (0.44-0.81)
	Ever-regular	17	1.00 (0.62-1.61)	38	0.58 (0.42-0.80)	50	0.66 (0.50-0.87)
Oesophagus	Never-regular	33	1.00 (0.71-1.41)	130	0.81 (0.68-0.96)	105	0.68 (0.56-0.83)
	Ever-regular	46	1.00 (0.75-1.34)	116	0.65 (0.54-0.78)*	116	0.60 (0.50-0.73)**
Liver	Never-regular	31	1.00 (0.70-1.43)	144	0.83 (0.71-0.98)	167	0.87 (0.74-1.01)
	Ever-regular	43	1.00 (0.74-1.35)	131	0.77 (0.65-0.91)	135	0.73 (0.61-0.86)
Colon-rectum	Never-regular	20	1.00 (0.64-1.55)	124	1.04 (0.87-1.24)	133	0.94 (0.80-1.12)
	Ever-regular	24	1.00 (0.67-1.49)	125	1.33 (1.12-1.59)	130	1.24 (1.04-1.47)
Lung	Never-regular	31	1.00 (0.70-1.42)	245	1.32 (1.17-1.50)	282	1.30 (1.15-1.46)
	Ever-regular	65	1.00 (0.78-1.28)	245	0.95 (0.84-1.08)	267	0.95 (0.84-1.07)
Stomach	Never-regular	36	1.00 (0.72-1.39)	175	0.92 (0.80-1.07)	180	0.91 (0.79-1.06)
	Ever-regular	34	1.00 (0.71-1.40)	142	1.08 (0.92-1.28)	158	1.10 (0.94-1.29)
Other cancers of known sites	Never-regular	51	1.00 (0.76-1.32)	267	0.88 (0.78-0.99)	306	0.87 (0.78-0.98)
	Ever-regular	63	1.00 (0.78-1.28)	225	0.91 (0.80-1.04)	273	0.98 (0.87-1.10)
IARC alcohol-related	Never-regular	92	1.00 (0.81-1.23)	434	0.85 (0.78-0.94)	435	0.78 (0.71-0.86)*
	Ever-regular	127	1.00 (0.84-1.19)	395	0.80 (0.72-0.88)*	417	0.76 (0.69-0.84)**
All cancers[a]	Never-regular	190	1.00 (0.87-1.15)	1018	0.94 (0.89-1.00)	1095	0.90 (0.85-0.96)
	Ever-regular	277	1.00 (0.89-1.13)	922	0.85 (0.80-0.91)*	1007	0.84 (0.79-0.90)*

HR, hazard ratio; CI, confidence interval; IARC, International Agency for Research on Cancer.
Cox models were stratified by age-at-risk and study area, and adjusted for 12 genomic principal components.
Never-regular drinkers included abstainers and occasional drinkers; ever-regular drinkers included ex-regular and current regular drinkers.
HRs were presented with group-specific 95% CIs to enable comparison between any two genotypes within the same drinking status.
[a] All cancers included ill-defined neoplasm and are patient-based.
**P*<0.05; ***P*<0.01, for association comparing the marked genotype versus GG genotype within the same drinking status.

Figure S2. Adjusted HRs for total and site-specific cancers per 280 g/week higher usual alcohol intake in male current regular drinkers, stratified by *ALDH2*-rs671 genotype

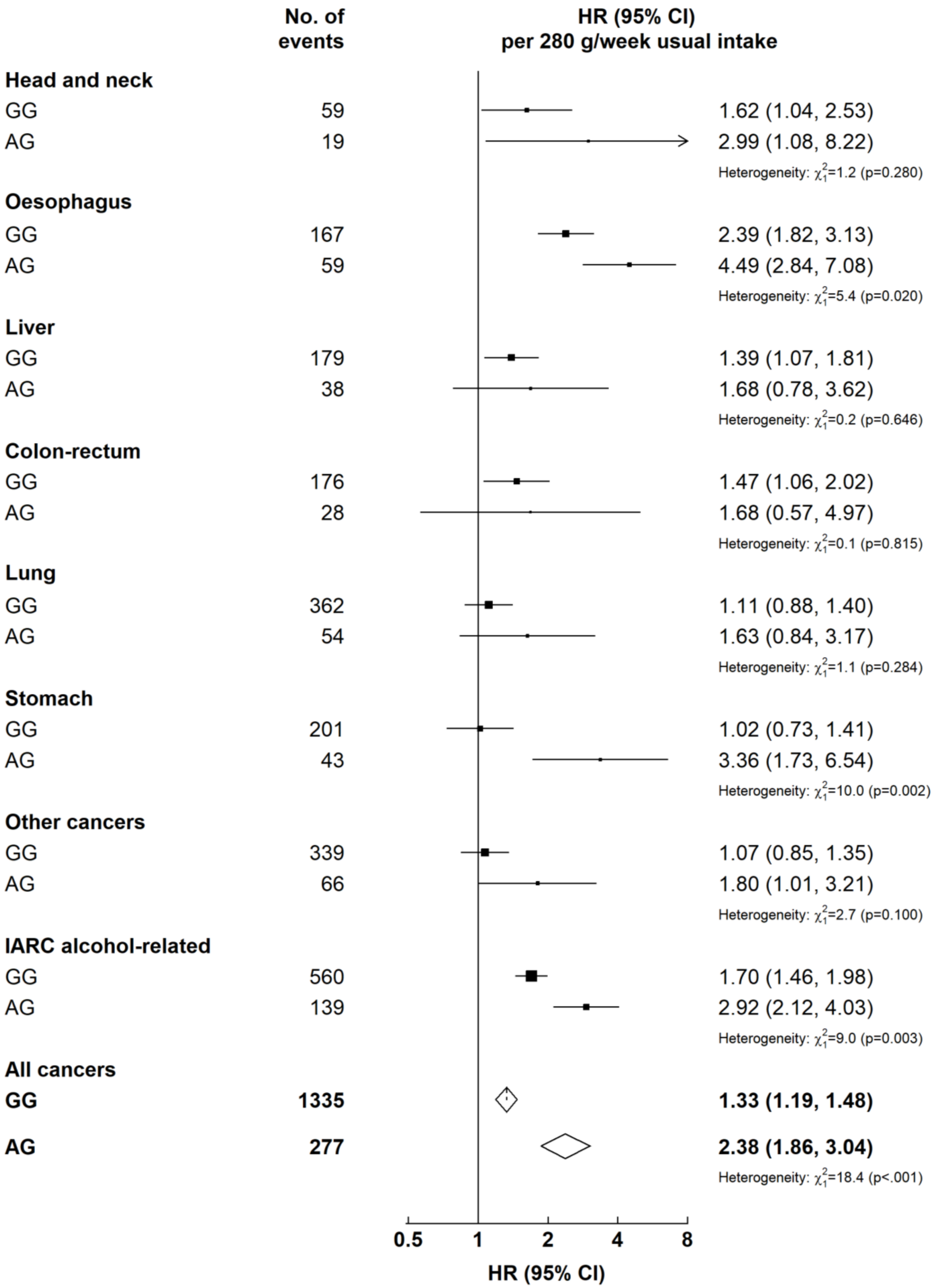

Cox models were stratified by age-at-risk and study area, and adjusted for 12 genomic principal components, education, household income, smoking status, physical activity, fresh fruit intake, body mass index, and family history of cancer. Each solid square represents an HR. The size of each box is inversely proportional to the variance of the log HR and the error bars indicate 95% CI. Open diamonds represent the overall HRs for all cancers. AA individuals were excluded as few of them drank (n=28). HR, hazard ratio; CI, confidence interval; IARC, International Agency for Research on Cancer.

Figure S3. Associations of *ADH1B*-rs1229984 genotypes with risks of total and site-specific cancers at different usual intake levels of alcohol, in male current regular drinkers

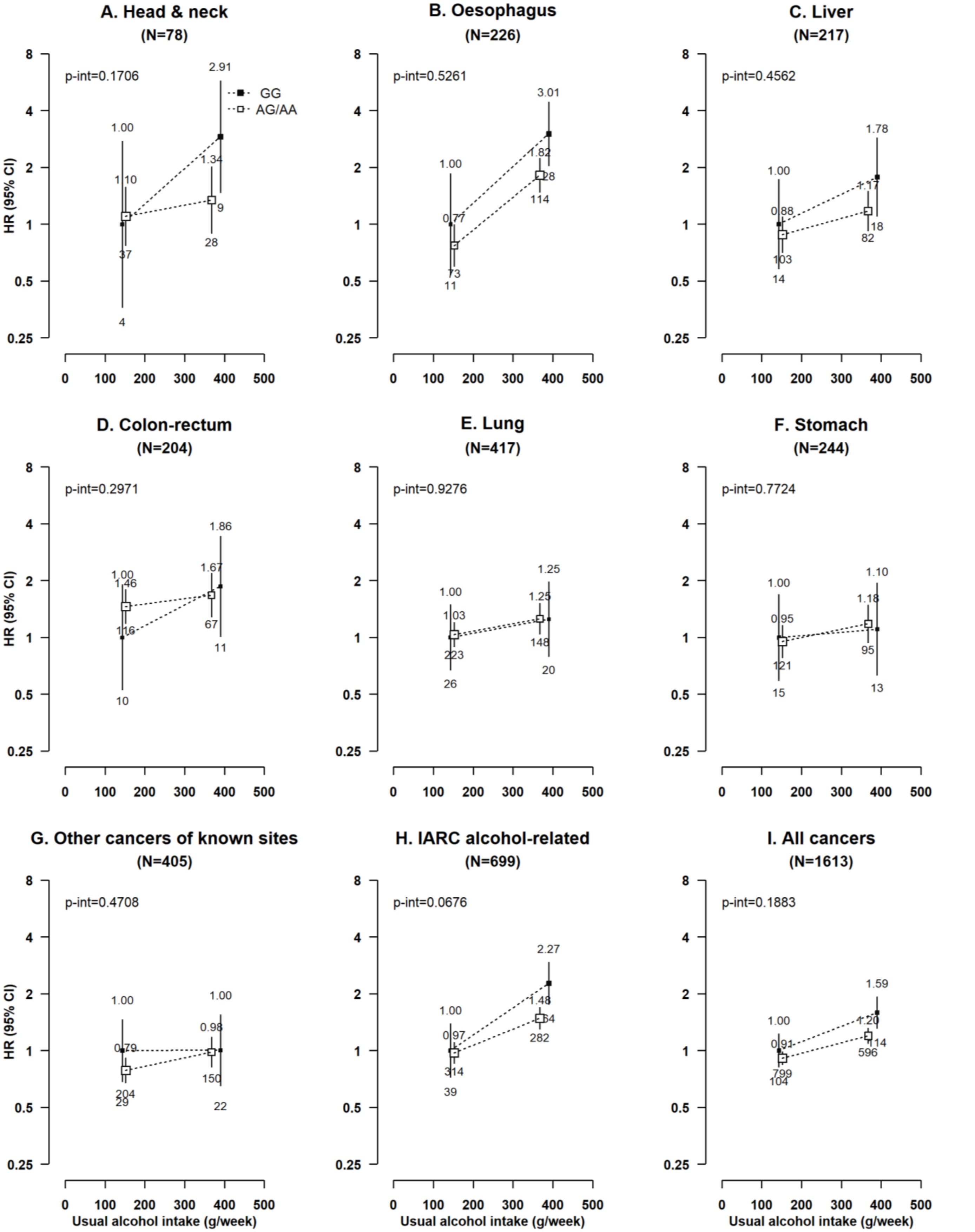

Cox models were stratified by age-at-risk and study area, and adjusted for 12 genomic principal components, education, household income, smoking status, physical activity, fresh fruit intake, body mass index, and family history of cancer. Each box represents HR with the area inversely proportional to the variance of the group-specific log hazard. The vertical lines indicate group-specific 95% CIs. The numbers above the error bars are point estimates for HRs, and the numbers below are number of events. Solid boxes denote *ADH1B*-rs1229984 GG genotype and open boxes denote *ADH1B*-rs1229984 AG/AA genotypes. Alcohol intake, separately in *ADH1B*-rs1229984 GG and AG/AA drinkers, was classified based on baseline consumption of <280 and ≥280 g/week. IARC, International Agency for Research on Cancer; HR, hazard ratio; CI, confidence interval.

Figure S4. Adjusted HRs for total and site-specific cancers per 280 g/week higher usual alcohol intake in male current regular drinkers, stratified by *ADH1B*-rs1229984 genotype

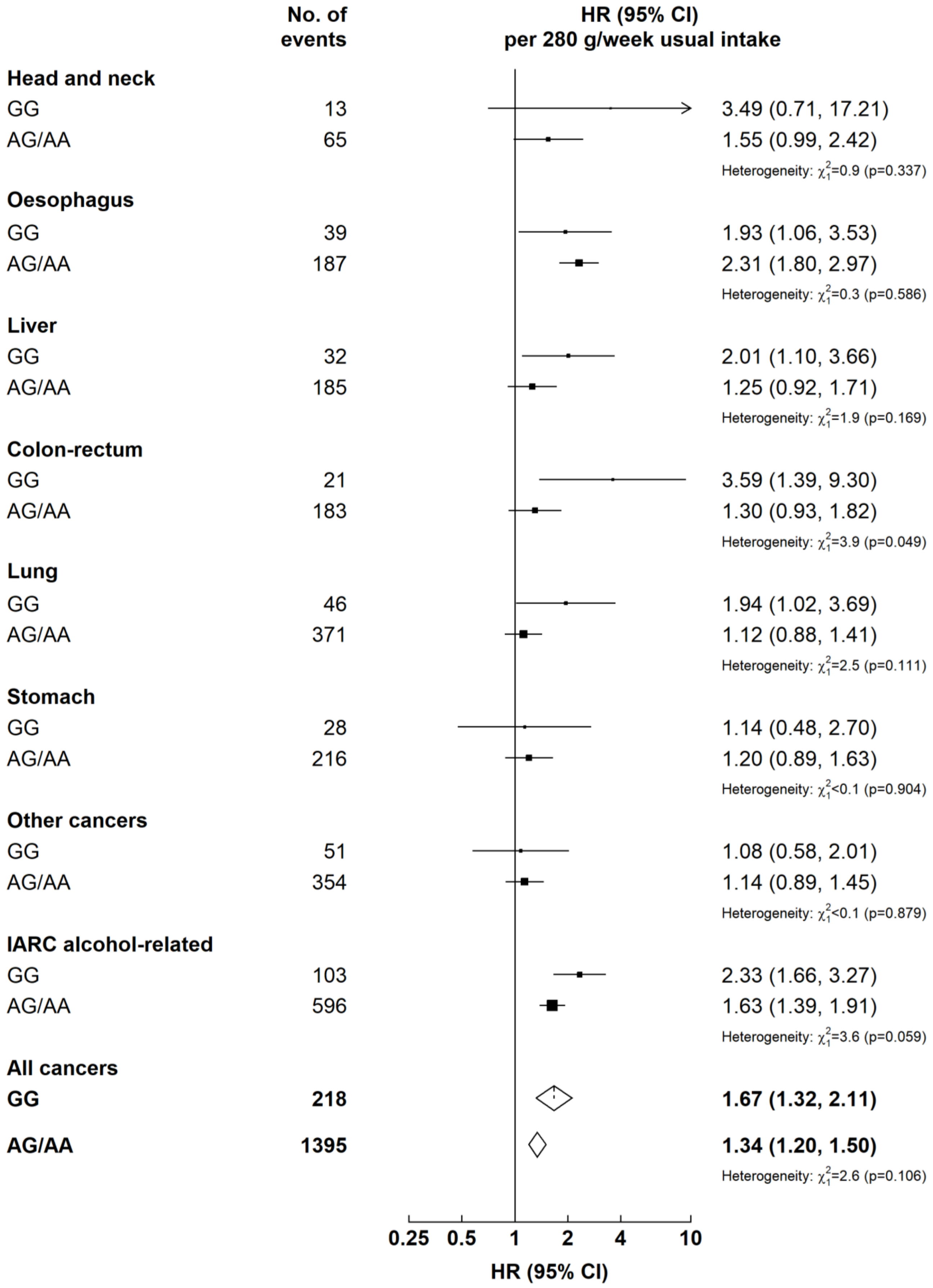

HR, hazard ratio; CI, confidence interval; IARC, International Agency for Research on Cancer. Conventions are as in Figure S2.

Table S6. Adjusted HRs for total and site-specific cancers associated with genotypes of both *ADH1B*-rs1229984 and *ALDH2*-rs671, in men

		***ADH1B*-rs1229984**							
		**GG**		**AG**		**AA**			
	***ALDH2*-rs671**	**N**	**HR (95% CI)**	**N**	**HR (95% CI)**	**N**	**HR (95% CI)**	***P*association[b]**	***P*interaction[c]**
Upper aerodigestive tract	GG	60	1.00 (0.78-1.29)	216	0.77 (0.67-0.88)	208	0.71 (0.62-0.82)*	.	.
	AG	39	1.47 (1.07-2.01)	95	0.78 (0.63-0.95)	97	0.71 (0.58-0.87)*	.	.
	AA	3	0.93 (0.30-2.89)	9	0.59 (0.31-1.14)	4	0.25 (0.10-0.68)**	0.0006	0.2958
Liver	GG	44	1.00 (0.74-1.35)	183	0.89 (0.77-1.03)	184	0.82 (0.71-0.94)	.	.
	AG	27	1.21 (0.83-1.76)	85	0.79 (0.64-0.98)	106	0.85 (0.70-1.03)	.	.
	AA	3	0.88 (0.28-2.73)	7	0.43 (0.20-0.90)*	12	0.71 (0.40-1.26)	0.3405	0.5939
Colon-rectum	GG	26	1.00 (0.68-1.47)	157	1.26 (1.07-1.47)	170	1.19 (1.02-1.38)	.	.
	AG	15	1.06 (0.64-1.76)	85	1.23 (1.00-1.53)	82	1.02 (0.82-1.27)	.	.
	AA	3	1.56 (0.50-4.84)	7	0.66 (0.32-1.39)	11	0.95 (0.52-1.72)	0.6229	0.5873
Lung	GG	67	1.00 (0.79-1.27)	288	0.88 (0.78-0.99)	312	0.85 (0.76-0.95)	.	.
	AG	27	0.73 (0.50-1.07)	179	0.99 (0.86-1.15)	208	0.98 (0.85-1.12)	.	.
	AA	2	0.36 (0.09-1.44)	23	0.79 (0.53-1.19)	29	0.96 (0.66-1.38)	0.4049	0.1831
Stomach	GG	43	1.00 (0.74-1.35)	212	1.07 (0.94-1.23)	212	1.02 (0.89-1.16)	.	.
	AG	24	1.22 (0.82-1.82)	96	1.04 (0.85-1.27)	115	1.12 (0.93-1.35)	.	.
	AA	3	1.20 (0.39-3.73)	9	0.73 (0.38-1.40)	11	0.95 (0.52-1.72)	0.9456	0.8031
Other cancers of known sites	GG	67	1.00 (0.79-1.27)	301	0.92 (0.82-1.04)	352	0.96 (0.86-1.07)	.	.
	AG	37	1.01 (0.73-1.40)	173	0.98 (0.84-1.14)	201	0.96 (0.84-1.11)	.	.
	AA	10	1.91 (1.03-3.56)	18	0.65 (0.41-1.03)	26	0.89 (0.61-1.31)	0.4003	0.2188
IARC alcohol-related	GG	129	1.00 (0.84-1.19)	543	0.89 (0.82-0.97)	544	0.82 (0.76-0.90)*	.	.
	AG	81	1.28 (1.03-1.59)	264	0.88 (0.78-0.99)	281	0.81 (0.72-0.92)	.	.
	AA	9	1.04 (0.54-2.01)	22	0.51 (0.34-0.78)**	27	0.59 (0.41-0.87)*	0.0006	0.3544
All cancers[a]	GG	290	1.00 (0.89-1.12)	1220	0.88 (0.83-0.93)	1282	0.84 (0.80-0.89)**	.	.
	AG	154	1.04 (0.89-1.22)	653	0.92 (0.85-0.99)	732	0.89 (0.83-0.96)	.	.
	AA	23	1.12 (0.75-1.69)	67	0.63 (0.49-0.80)***	88	0.79 (0.64-0.98)	0.0043	0.3327

HR, hazard ratio; CI, confidence interval; IARC, International Agency for Research on Cancer.
Cox models were stratified by age-at-risk and study area, and adjusted for 12 genomic principal components.
HRs were presented with group-specific 95% CIs to enable comparison between any two genotypes.
The corresponding baseline mean alcohol intake (g/week) for each combined genotype category of ***ALDH2*-rs671**/*ADH1B*-rs1229984 are: 178.7 for **GG**/GG, 137.6 for **GG**/AG, 133.3 for **GG**/AA, 79.2 for **AG**/GG, 35.5 for **AG**/AG, 27.4 for **AG**/AA, 4.7 for **AA**/GG, 2.0 for **AA**/AG, 1.6 for **AA**/AA, assuming an intake of 0 g/week to baseline non-drinkers, and 5 g/week to baseline occasional drinkers.
[a] All cancers included ill-defined neoplasm and are patient-based.
[b] P for association for the two-way joint-effect variable in the model.
[c] P for interaction obtained from likelihood ratio tests comparing two models with and without the product term.
**P*<0.05; ***P*<0.01, for association comparing the marked group with the reference group.

Figure S5. Adjusted HRs for total and site-specific cancers associated with genotypes separately for *ALDH2*-rs671 and *ADH1B*-rs1229984 in men, after further adjustment for conventional cancer-related risk factors

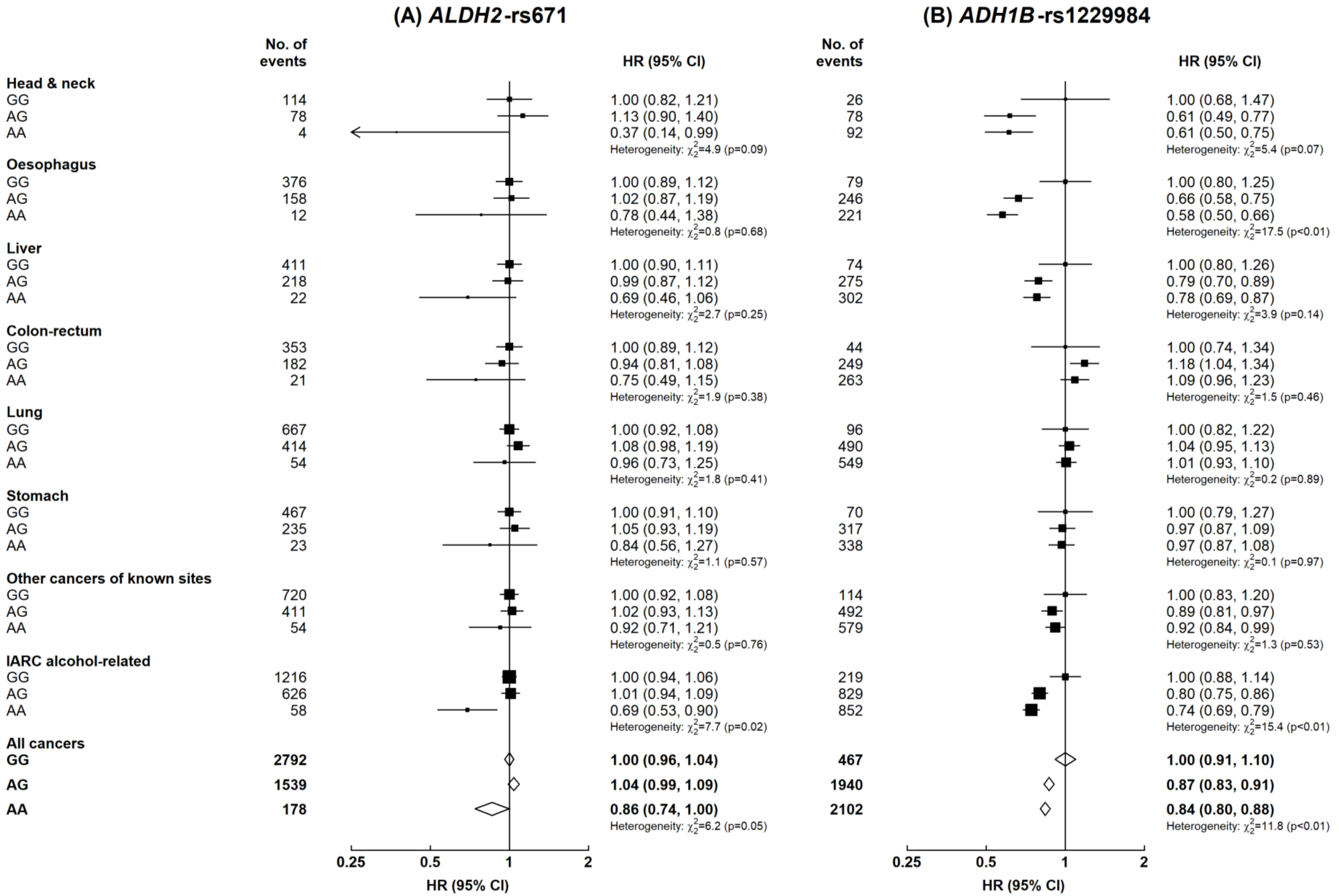

Cox models were stratified by age-at-risk and study area, and adjusted for 12 genomic principal components, education, household income, smoking status, physical activity, fresh fruit intake, body mass index, family history of cancer, and hepatitis B surface antigen. Each solid square represents HR with the area inversely proportional to the "floated" variance of the group-specific log hazard. The horizontal lines indicate group-specific 95% CIs. Open diamonds represent the overall HRs for all cancers. IARC, International Agency for Research on Cancer; HR, hazard ratio; CI, confidence interval.

Figure S6. Adjusted HRs for total and site-specific cancers associated with genotypes separately for *ALDH2*-rs671 and *ADH1B*-rs1229984 in men, estimated by area-adjusted and area-stratified analyses

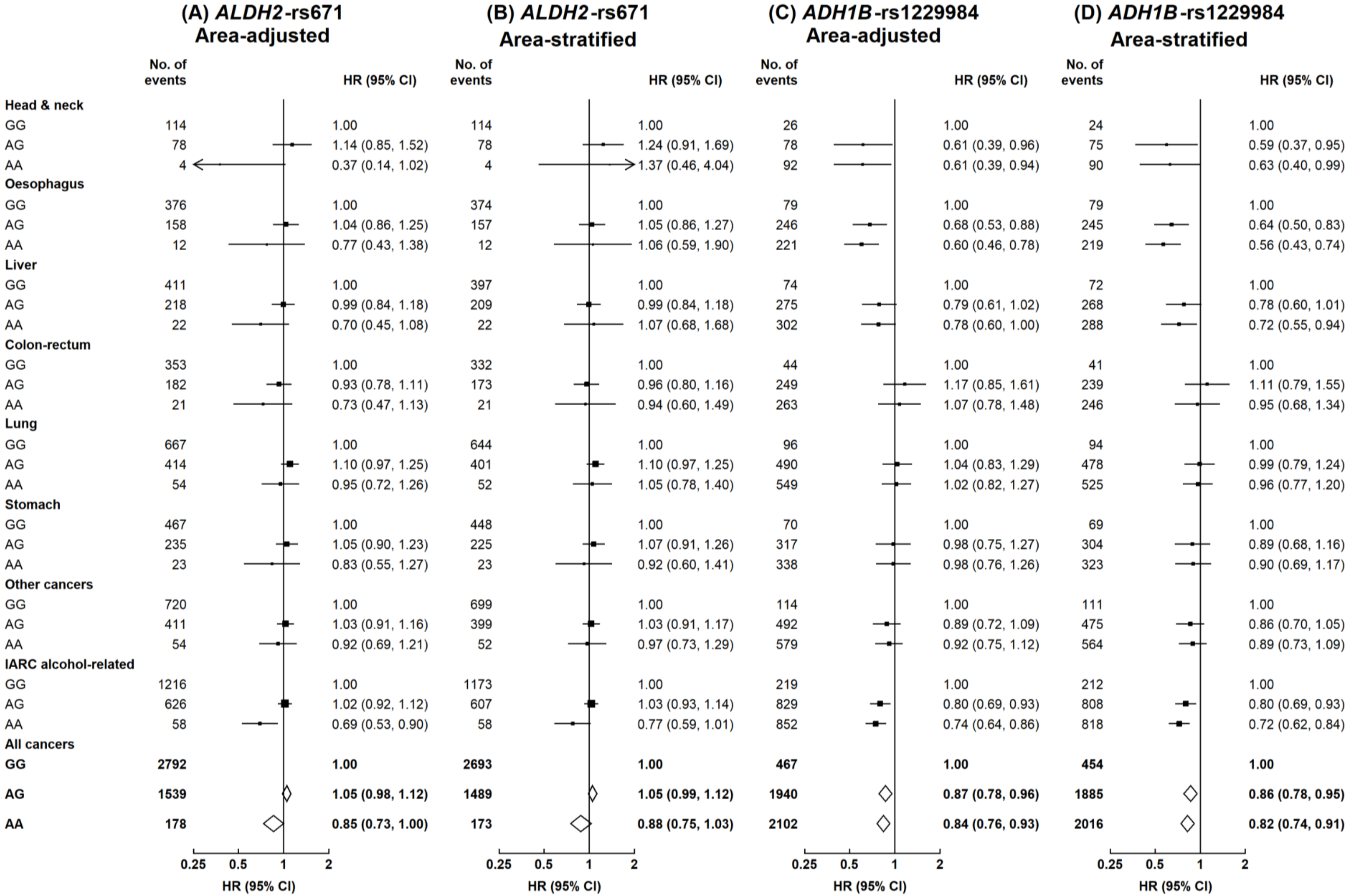

(A, C) represented findings from area-adjusted analysis (main analysis), where HRs were estimated using Cox models stratified by age-at-risk and study area, and adjusted for 12 genomic principal components, in all men. (B, D) represented findings from area-stratified analysis (sensitivity analysis), where HRs were estimated by calculating inverse variance-weighted estimates of within-area estimates, which were calculated from Cox models stratified by age-at-risk and adjusted for the corresponding principal components within study area. Each solid square represents HR with the area inversely proportional to the variance of the log HR. The horizontal lines indicate 95% CIs. Open diamonds represent the overall HRs for all cancers. IARC, International Agency for Research on Cancer; HR, hazard ratio; CI, confidence interval.

Figure S7. Associations of *ALDH2*-rs1671 genotypes with risks of total and site-specific cancers at different usual intake levels of alcohol in male current regular drinkers, after excluding individuals with prior cancer at baseline

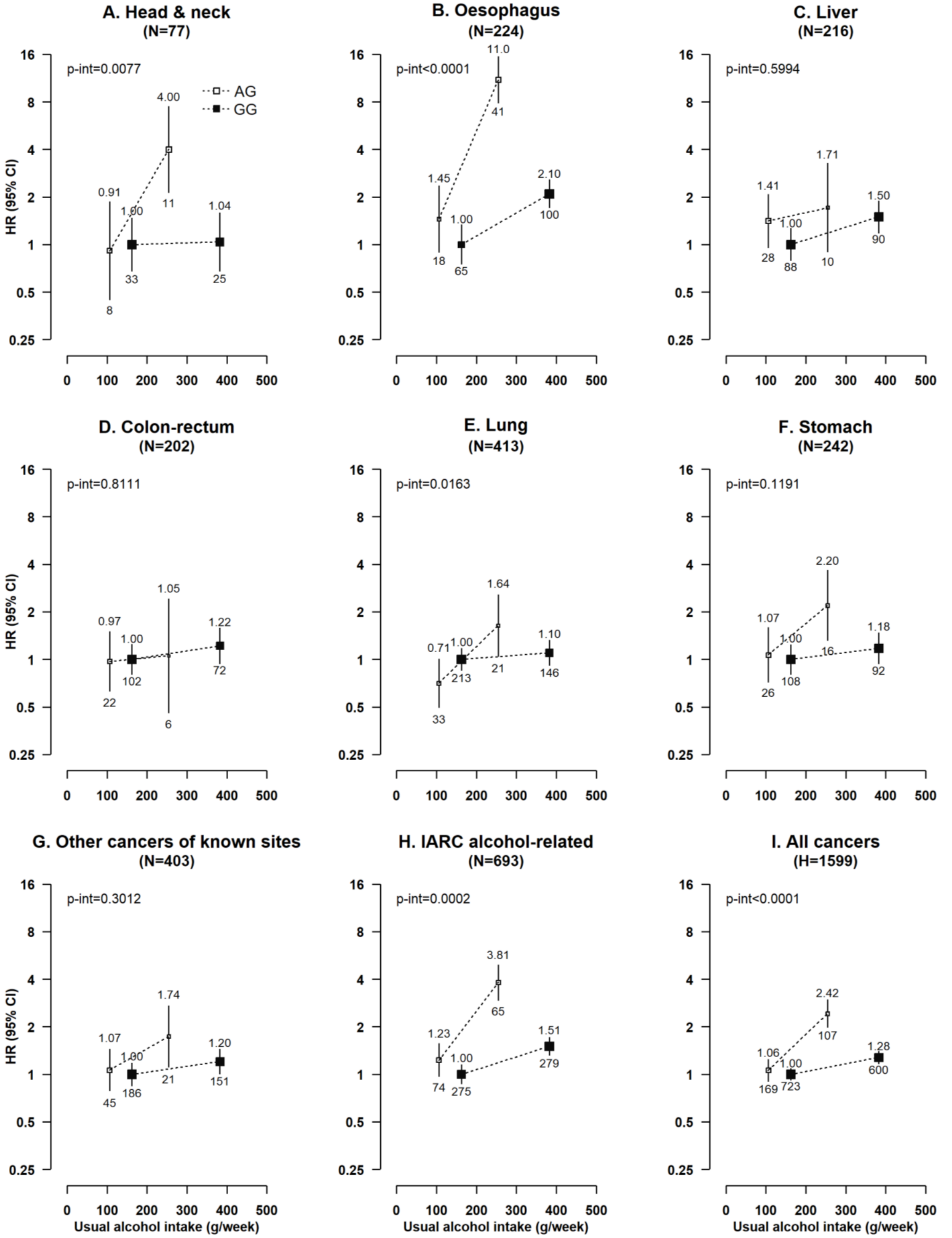

Solid boxes denote *ALDH2*-rs671 GG genotype and open boxes denote *ALDH2*-rs671 AG genotype. Alcohol intake, separately in *ALDH2*-rs671 GG and AG drinkers, was classified based on baseline consumption of <280 and ≥280 g/week. HR, hazard ratio; CI, confidence interval; IARC, International Agency for Research on Cancer. Conventions are as in Figure S3.

Figure S8. Associations of *ALDH2*-rs1671 genotypes with risks of total and site-specific cancers at different usual intake levels of alcohol in male current regular drinkers, after further adjustment for hepatitis B infection status

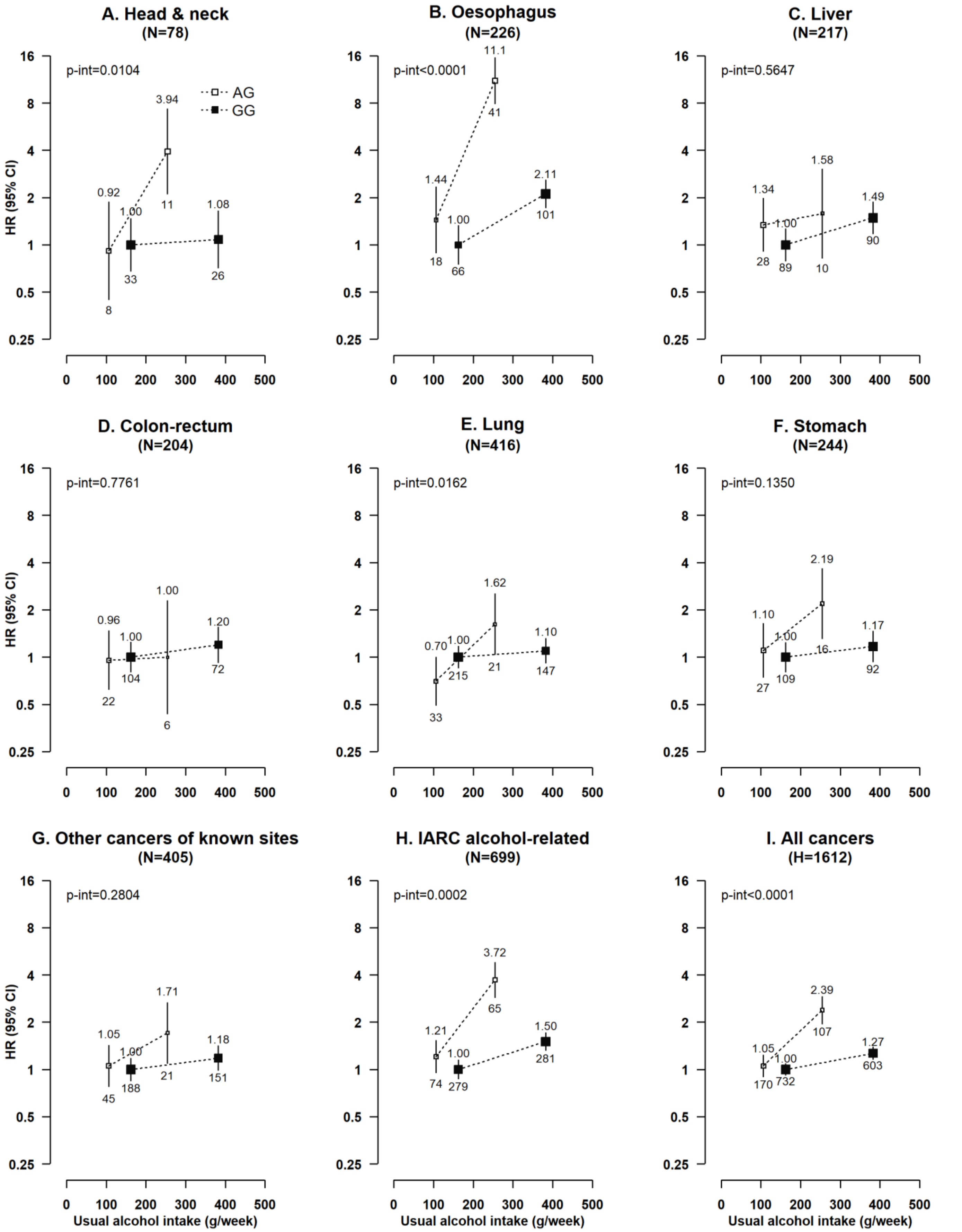

Cox models were stratified by age-at-risk and study area, and adjusted for 12 genomic principal components, education, household income, smoking status, physical activity, fresh fruit intake, body mass index, family history of cancer, and hepatitis B surface antigen. HR, hazard ratio; CI, confidence interval; IARC, International Agency for Research on Cancer. Conventions are as in Figure S7.

Table S7. Adjusted HRs for total and site-specific cancers associated with genotypes of *ALDH2*-rs671 and *ADH1B*-rs1229984, stratified by sex

		ALDH2-rs671					*ADH1B*-rs1229984				
		Men		Women			Men		Women		
	Genotype	N	HR (95%CI)	N	HR (95%CI)	$P_{heterogeneity}$[b]	N	HR (95%CI)	N	HR (95%CI)	$P_{heterogenetiy}$[b]
Head and neck	GG	114	1.00 (0.82-1.21)	71	1.00 (0.79-1.27)	--	26	1.00 (0.68-1.47)	5	1.00 (0.42-2.40)	--
	AG	78	1.14 (0.91-1.41)	40	0.94 (0.69-1.28)	0.445	78	0.61 (0.49-0.77)	55	2.42 (1.86-3.15)	0.008
	AA	4	0.37 (0.14-1.00)	9	1.35 (0.70-2.60)	0.040	92	0.61 (0.50-0.75)	60	2.19 (1.69-2.82)	0.013
Oesophagus	GG	376	1.00 (0.89-1.12)	184	1.00 (0.86-1.17)	--	79	1.00 (0.80-1.25)	31	1.00 (0.70-1.42)	--
	AG	158	1.04 (0.89-1.21)	42	0.60 (0.45-0.81)	0.006	246	0.68 (0.60-0.77)	97	0.83 (0.68-1.02)	0.410
	AA	12	0.77 (0.44-1.36)	6	0.80 (0.36-1.79)	0.940	221	0.60 (0.53-0.69)	104	0.85 (0.70-1.03)	0.151
Liver	GG	411	1.00 (0.90-1.11)	213	1.00 (0.87-1.15)	--	74	1.00 (0.80-1.26)	40	1.00 (0.73-1.36)	--
	AG	218	0.99 (0.87-1.13)	105	0.97 (0.80-1.17)	0.844	275	0.79 (0.70-0.88)	156	0.91 (0.78-1.06)	0.513
	AA	22	0.70 (0.46-1.07)	25	1.61 (1.08-2.39)	0.007	302	0.78 (0.69-0.87)	147	0.76 (0.65-0.90)	0.919
Colon-rectum	GG	353	1.00 (0.90-1.12)	352	1.00 (0.90-1.12)	--	44	1.00 (0.74-1.34)	52	1.00 (0.76-1.31)	--
	AG	182	0.93 (0.81-1.07)	202	1.09 (0.95-1.24)	0.228	249	1.17 (1.03-1.32)	249	1.09 (0.97-1.24)	0.770
	AA	21	0.73 (0.47-1.12)	24	0.86 (0.58-1.29)	0.584	263	1.07 (0.95-1.21)	277	1.07 (0.95-1.21)	0.992
Breast	GG	--	--	545	1.00 (0.91-1.09)	--	--	--	72	1.00 (0.79-1.26)	--
	AG	--	--	281	1.01 (0.90-1.14)	--	--	--	365	1.17 (1.06-1.30)	--
	AA	--	--	45	1.14 (0.85-1.53)	--	--	--	434	1.23 (1.12-1.35)	--
Lung	GG	667	1.00 (0.92-1.08)	503	1.00 (0.91-1.10)	--	96	1.00 (0.82-1.22)	65	1.00 (0.78-1.28)	--
	AG	414	1.10 (1.00-1.21)	297	1.14 (1.02-1.28)	0.709	490	1.04 (0.95-1.14)	359	1.26 (1.14-1.40)	0.266
	AA	54	0.95 (0.73-1.24)	37	1.00 (0.73-1.39)	0.816	549	1.02 (0.94-1.11)	413	1.29 (1.17-1.43)	0.178
Stomach	GG	467	1.00 (0.91-1.10)	228	1.00 (0.87-1.15)	--	70	1.00 (0.79-1.26)	37	1.00 (0.72-1.38)	--
	AG	235	1.05 (0.93-1.19)	131	1.17 (0.99-1.38)	0.446	317	0.98 (0.88-1.09)	165	1.05 (0.90-1.23)	0.739
	AA	23	0.84 (0.55-1.26)	23	1.49 (0.99-2.25)	0.061	338	0.98 (0.88-1.09)	180	1.03 (0.89-1.20)	0.800
Other cancers of known sites	GG	720	1.00 (0.93-1.08)	1101	1.00 (0.94-1.06)	--	114	1.00 (0.83-1.20)	186	1.00 (0.87-1.15)	--
	AG	411	1.03 (0.93-1.13)	627	1.10 (1.01-1.18)	0.426	492	0.89 (0.81-0.97)	744	0.92 (0.86-0.99)	0.747
	AA	54	0.92 (0.70-1.20)	89	1.07 (0.87-1.32)	0.383	579	0.92 (0.84-0.99)	887	0.97 (0.91-1.04)	0.652
IARC alcohol-related	GG	1216	1.00 (0.94-1.06)	1332	1.00 (0.94-1.06)	--	219	1.00 (0.88-1.14)	199	1.00 (0.87-1.15)	--
	AG	626	1.02 (0.94-1.10)	652	0.97 (0.90-1.05)	0.528	829	0.80 (0.75-0.86)	903	1.06 (0.99-1.13)	0.010
	AA	58	0.69 (0.53-0.90)	106	1.12 (0.92-1.35)	0.005	852	0.75 (0.70-0.80)	988	1.03 (0.97-1.10)	0.003
All cancers[a]	GG	2792	1.00 (0.96-1.04)	2997	1.00 (0.96-1.04)	--	467	1.00 (0.91-1.10)	461	1.00 (0.91-1.10)	--
	AG	1539	1.05 (1.00-1.10)	1590	1.05 (1.00-1.10)	0.963	1940	0.87 (0.83-0.91)	2045	1.03 (0.99-1.08)	0.016
	AA	178	0.86 (0.74-0.99)	243	1.12 (0.99-1.27)	0.008	2102	0.84 (0.81-0.88)	2324	1.04 (1.00-1.09)	0.003

HR, hazard ratio; CI, confidence interval; IARC, International Agency for Research on Cancer.
Cox models were stratified by age-at-risk and study area, and adjusted for 12 genomic principal components.
HRs were presented with group-specific 95% CIs to enable comparison between any two genotypes of the same genetic variant within the same sex.
[a] All cancers included ill-defined neoplasm and are patient-based.
[b] P for heterogeneity in the HRs between men and women (assessed by chi-square tests for heterogeneity applied to the log HRs and their SEs).

Table S8. Adjusted HRs for total and site-specific cancers associated with *ALDH2*-rs671 genotypes in women, stratified by drinking status

		ALDH2-rs671					
		GG		AG		AA	
	**Drinking status**	**N**	**HR (95% CI)**	**N**	**HR (95% CI)**	**N**	**HR (95% CI)**
Head and neck	Never-regular	68	1.00 (0.78-1.28)	40	0.95 (0.70-1.29)	9	1.35 (0.70-2.61)
	Ever-regular	3	1.00[a]	0	--	0	--
Oesophagus	Never-regular	172	1.00 (0.85-1.17)	42	0.63 (0.47-0.85)**	6	0.84 (0.37-1.87)
	Ever-regular	12	1.00[a]	0	--	0	--
Liver	Never-regular	201	1.00 (0.86-1.16)	102	0.95 (0.79-1.16)	25	1.62 (1.09-2.41)*
	Ever-regular	12	1.00[a]	3	3.41 (0.80-14.59)	0	--
Colon-rectum	Never-regular	332	1.00 (0.89-1.12)	201	1.11 (0.97-1.27)	24	0.88 (0.59-1.31)
	Ever-regular	20	1.00[a]	1	0.37 (0.05-2.87)	0	--
Breast	Never-regular	520	1.00 (0.91-1.10)	277	1.02 (0.91-1.14)	45	1.14 (0.85-1.54)
	Ever-regular	25	1.00[a]	4	0.80 (0.26-2.45)	0	--
Lung	Never-regular	482	1.00 (0.91-1.10)	295	1.14 (1.02-1.28)	37	1.00 (0.72-1.38)
	Ever-regular	21	1.00[a]	2	0.71 (0.16-3.17)	0	--
Stomach	Never-regular	218	1.00 (0.87-1.15)	129	1.16 (0.98-1.37)	23	1.49 (0.99-2.25)
	Ever-regular	10	1.00[a]	2	2.56 (0.45-14.68)	0	--
Other cancers of known sites	Never-regular	1053	1.00 (0.94-1.07)	620	1.10 (1.02-1.19)	89	1.08 (0.87-1.33)
	Ever-regular	48	1.00[a]	7	0.98 (0.43-2.25)	0	--
IARC alcohol-related	Never-regular	1263	1.00 (0.94-1.06)	644	0.98 (0.91-1.06)	106	1.13 (0.93-1.37)
	Ever-regular	69	1.00[a]	8	0.82 (0.39-1.76)	0	--
All cancers[b]	Never-regular	2857	1.00 (0.96-1.04)	1572	1.05 (1.00-1.10)	243	1.13 (0.99-1.28)
	Ever-regular	140	1.00[a]	18	0.92 (0.55-1.53)	0	--

HR, hazard ratio; CI, confidence interval; IARC, International Agency for Research on Cancer.
Cox models were stratified by age-at-risk and study area, and adjusted for 12 genomic principal components.
Never-regular drinkers included abstainers and occasional drinkers; ever-regular drinkers included ex-regular and current regular drinkers.
Among never-regular drinkers, HRs were presented with group-specific 95% CIs to enable comparison between any two genotypes.
[a] Among ever-regular drinkers *ALDH2*-rs671 AA individuals were excluded from the analysis due to small numbers, and HRs were presented with conventional 95% CIs for two-way comparison of AG vs. GG.
[b] All cancers included ill-defined neoplasm and are patient-based.
**P*<0.05; ***P*<0.01, for association comparing the marked genotype versus GG genotype within the same drinking status.

Table S9. Adjusted HRs for total and site-specific cancers associated with *ADH1B*-rs1229984 genotypes in women, stratified by drinking status

		ADH1B-rs1229984					
		GG		AG		AA	
	Drinking status	N	HR (95% CI)	N	HR (95% CI)	N	HR (95% CI)
Head and neck	Never-regular	5	1.00 (0.42-2.40)	54	2.34 (1.79-3.05)	58	2.07 (1.60-2.68)
	Ever-regular	0	--	1	--	2	--
Oesophagus	Never-regular	27	1.00 (0.68-1.46)	93	0.90 (0.74-1.11)	100	0.92 (0.76-1.12)
	Ever-regular	4	1.00 (0.31-3.24)	4	0.23 (0.08-0.65)	4	0.19 (0.06-0.61)*
Liver	Never-regular	37	1.00 (0.72-1.38)	151	0.93 (0.80-1.10)	140	0.77 (0.65-0.91)
	Ever-regular	3	1.00 (0.27-3.67)	5	0.71 (0.30-1.72)	7	0.88 (0.37-2.10)
Colon-rectum	Never-regular	49	1.00 (0.76-1.32)	240	1.10 (0.97-1.25)	268	1.08 (0.96-1.22)
	Ever-regular	3	1.00 (0.31-3.27)	9	0.74 (0.37-1.48)	9	0.71 (0.35-1.43)
Breast	Never-regular	71	1.00 (0.79-1.26)	350	1.12 (1.01-1.25)	421	1.19 (1.08-1.31)
	Ever-regular	1	1.00 (0.14-7.28)	15	4.69 (2.77-7.95)	13	4.11 (2.37-7.13)
Lung	Never-regular	60	1.00 (0.78-1.29)	351	1.32 (1.19-1.46)*	403	1.34 (1.22-1.48)*
	Ever-regular	5	1.00 (0.40-2.52)	8	0.53 (0.26-1.09)	10	0.67 (0.35-1.28)
Stomach	Never-regular	37	1.00 (0.72-1.38)	159	1.01 (0.86-1.18)	174	0.99 (0.85-1.14)
	Ever-regular	0	--	6	--	6	--
Other cancers of known sites	Never-regular	177	1.00 (0.86-1.16)	721	0.93 (0.86-1.00)	864	0.98 (0.91-1.04)
	Ever-regular	9	1.00 (0.51-1.96)	23	0.65 (0.43-0.99)	23	0.69 (0.45-1.05)
IARC alcohol-related	Never-regular	188	1.00 (0.87-1.15)	869	1.06 (1.00-1.14)	956	1.04 (0.97-1.11)
	Ever-regular	11	1.00 (0.54-1.85)	34	1.00 (0.71-1.42)	32	0.92 (0.64-1.31)
All cancers[a]	Never-regular	440	1.00 (0.91-1.10)	1974	1.03 (0.99-1.08)	2258	1.04 (1.00-1.09)
	Ever-regular	21	1.00 (0.65-1.55)	71	1.00 (0.79-1.28)	66	0.94 (0.73-1.21)

HR, hazard ratio; CI, confidence interval; IARC, International Agency for Research on Cancer.
Cox models were stratified by age-at-risk and study area, and adjusted for 12 genomic principal components.
Never-regular drinkers included abstainers and occasional drinkers; ever-regular drinkers included ex-regular and current regular drinkers.
HRs were presented with group-specific 95% CIs to enable comparison between any two genotypes within the same drinking status.
[a] All cancers included ill-defined neoplasm and are patient-based.
*P<0.05; **P<0.01, for association comparing the marked genotype versus GG genotype within the same drinking status.

Table S10. Adjusted HRs for total and IARC alcohol-related cancers associated with both alcohol intake and genotypes of *ALDH2*-rs671 and *ADH1B*-rs1229984, in female current regular drinkers

		<70 g/week		70+ g/week		
	**Genotype**	**N**	**HR (95% CI)**	**N**	**HR (95% CI)**	$P_{interaction}$[c]
***ALDH2*-rs671**[a]						
IARC alcohol-related cancers	GG	30	1.00 (0.66-1.51)	19	0.79 (0.46-1.37)	
	AG	3	0.48 (0.15-1.57)	3	2.17 (0.64-7.39)	0.0633
All cancers[b]	GG	51	1.00 (0.73-1.36)	44	0.94 (0.66-1.36)	
	AG	8	0.78 (0.37-1.65)	5	1.60 (0.63-4.08)	0.2380
***ADH1B*-rs1229984**						
IARC alcohol-related cancers	GG	4	1.00 (0.35-2.82)	3	0.86 (0.26-2.84)	
	AG/AA	29	1.09 (0.68-1.75)	19	1.07 (0.64-1.77)	0.8857
All cancers[b]	GG	6	1.00 (0.44-2.28)	8	1.48 (0.72-3.04)	
	AG/AA	53	1.40 (0.99-1.99)	41	1.36 (0.97-1.89)	0.4760

HR, hazard ratio; CI, confidence interval; IARC, International Agency for Research on Cancer.
Cox models were stratified by age-at-risk and study area, and adjusted for 12 genomic principal components, smoking, education, income, physical activity, fruit intake, body mass index, and family history of cancer.
HRs were presented with group-specific 95% CIs to enable comparison between any two genotype-alcohol groups.
Other cancer endpoints were not presented as there were less than three events in one of the exposure groups.
[a] *ALDH2*-rs671 AA individuals were excluded from the analysis due to small numbers.
[b] All cancers included ill-defined neoplasm and are patient-based.
[c] P for interaction obtained from likelihood ratio tests comparing two models with and without the product term.

Table S11. Adjusted HRs for selected cancers associated with both *ALDH2*-rs671 genotypes and usual intake levels of alcohol in male current regular drinkers, stratified by smoking status

			ALDH2-rs671								
			GG				AG				
			<280 g/week		280+ g/week		<280 g/week		280+ g/week		
	Smoking status	All N	N	HR (95% CI)	N	HR (95% CI)	N	HR (95% CI)	N	HR (95% CI)	$P_{interaction}$[b]
Upper aerodigestive tract	Never regular smokers	27	12	1.00 (0.56-1.78)	6	1.24 (0.55-2.77)	4	1.39 (0.52-3.74)	5	11.68 (4.67-29.20)	
	Ever regular smokers	268	85	1.35 (1.07-1.70)	118	2.47 (2.06-2.98)	21	1.64 (1.07-2.53)	44	10.53 (7.74-14.34)	0.6525
Lung	Never regular smokers	31	19	1.00 (0.63-1.58)	7	1.35 (0.64-2.84)	2	0.52 (0.13-2.08)	3	6.10 (1.93-19.31)	
	Ever regular smokers	385	196	2.44 (2.10-2.83)	140	2.79 (2.33-3.34)	31	1.77 (1.24-2.51)	18	3.76 (2.36-6.01)	0.3031
IARC alcohol-related	Never regular smokers	83	42	1.00 (0.73-1.36)	21	1.41 (0.92-2.17)	13	1.39 (0.80-2.42)	7	4.69 (2.19-10.03)	
	Ever regular smokers	616	237	1.28 (1.12-1.47)	260	1.95 (1.71-2.21)	61	1.53 (1.19-1.96)	58	4.76 (3.66-6.20)	0.8903
All cancers[a]	Never regular smokers	185	99	1.00 (0.82-1.22)	48	1.48 (1.11-1.97)	25	1.18 (0.79-1.75)	13	4.36 (2.50-7.58)	
	Ever regular smokers	1427	633	1.50 (1.38-1.63)	555	1.91 (1.74-2.08)	145	1.56 (1.32-1.83)	94	3.43 (2.79-4.21)	0.2777

HR, hazard ratio; CI, confidence interval; IARC, International Agency for Research on Cancer.
Cox models were stratified by age-at-risk and study area, and adjusted for 12 genomic principal components, education, household income, smoking status, physical activity, fresh fruit intake, body mass index, and family history of cancer.
HRs were presented with group-specific 95% CIs to enable comparison between any two groups.
[a] All cancers included ill-defined neoplasm and are patient-based.
[b] *P* for interaction obtained from likelihood ratio tests comparing two models with and without the three-way joint effect variable.

References

1. Cochrane J, Chen H, Conigrave KM, et al. Alcohol use in China. *Alcohol Alcohol*. 2003; **38**(6):537-42.

2. Pan R, Zhu M, Yu C, et al. Cancer incidence and mortality: A cohort study in China, 2008-2013. *Int J Cancer*. 2017; **141**(7):1315-23.

3. Millwood IY, Walters RG, Mei XW, et al. Conventional and genetic evidence on alcohol and vascular disease aetiology: a prospective study of 500 000 men and women in China. *Lancet*. 2019; **393**(10183):1831-42.

4. World Cancer Research Fund/American Institute for Cancer Research. Diet, Nutrition, Physical Activity and Cancer: a Global Perspective; 2018.

5. Millwood IY, Li L, Smith M, et al. Alcohol consumption in 0.5 million people from 10 diverse regions of China: prevalence, patterns and socio-demographic and health-related correlates. *Int J Epidemiol*. 2013; **42**(3):816-27.

6. Im PK, Millwood IY, Guo Y, et al. Patterns and trends of alcohol consumption in rural and urban areas of China: findings from the China Kadoorie Biobank. *BMC Public Health*. 2019; **19**(1):217.

7. IARC Working Group on the Evaluation of Carcinogenic Risks to Humans. Personal habits and indoor combustions. Volume 100 E. A review of human carcinogens. *IARC Monogr Eval Carcinog Risks Hum*. 2012; **100**(Pt E):1-538.

8. Easton DF, Peto J, Babiker AG. Floating absolute risk: an alternative to relative risk in survival and case-control analysis avoiding an arbitrary reference group. *Stat Med*. 1991; **10**(7):1025-35.

9. Clarke R, Shipley M, Lewington S, et al. Underestimation of risk associations due to regression dilution in long-term follow-up of prospective studies. *Am J Epidemiol*. 1999; **150**(4):341-53.

10. Im PK, Millwood IY, Kartsonaki C, et al. Alcohol drinking and risks of total and site-specific cancers in China: A 10-year prospective study of 0.5 million adults. *Int J Cancer*. 2021; **149**(3):522-34.

11. MacMahon S, Peto R, Cutler J, et al. Blood pressure, stroke, and coronary heart disease. Part 1, Prolonged differences in blood pressure: prospective observational studies corrected for the regression dilution bias. *Lancet*. 1990; **335**(8692):765-74.

12. Rosner B, Willett WC, Spiegelman D. Correction of logistic regression relative risk estimates and confidence intervals for systematic within-person measurement error. *Stat Med*. 1989; **8**(9):1051-73.
